# Cranial osteology of the ankylosaurian dinosaur formerly known as *Minmi* sp. (Ornithischia: Thyreophora) from the Lower Cretaceous Allaru Mudstone of Richmond, Queensland, Australia

**DOI:** 10.7717/peerj.1475

**Published:** 2015-12-08

**Authors:** Lucy G. Leahey, Ralph E. Molnar, Kenneth Carpenter, Lawrence M. Witmer, Steven W. Salisbury

**Affiliations:** 1 School of Biological Sciences, University of Queensland, Brisbane, Queensland, Australia; 2 University of California Museum of Paleontology, Berkeley, CA, USA; 3 Prehistoric Museum, Utah State University Eastern, Price, UT, USA; 4 Department of Biomedical Sciences, Heritage College of Osteopathic Medicine, Ohio University, Athens, OH, USA

**Keywords:** Dinosauria, Thyreophora, Eurypoda, Ankylosauria, Gondwana, Computed tomography, Nasal cavity, Braincase

## Abstract

*Minmi* is the only known genus of ankylosaurian dinosaur from Australia. Seven specimens are known, all from the Lower Cretaceous of Queensland. Only two of these have been described in any detail: the holotype specimen *Minmi paravertebra* from the Bungil Formation near Roma, and a near complete skeleton from the Allaru Mudstone on Marathon Station near Richmond, preliminarily referred to a possible new species of *Minmi*. The Marathon specimen represents one of the world’s most complete ankylosaurian skeletons and the best-preserved dinosaurian fossil from eastern Gondwana. Moreover, among ankylosaurians, its skull is one of only a few in which the majority of sutures have not been obliterated by dermal ossifications or surface remodelling. Recent preparation of the Marathon specimen has revealed new details of the palate and narial regions, permitting a comprehensive description and thus providing new insights cranial osteology of a basal ankylosaurian. The skull has also undergone computed tomography, digital segmentation and 3D computer visualisation enabling the reconstruction of its nasal cavity and endocranium. The airways of the Marathon specimen are more complicated than non-ankylosaurian dinosaurs but less so than derived ankylosaurians. The cranial (brain) endocast is superficially similar to those of other ankylosaurians but is strongly divergent in many important respects. The inner ear is extremely large and unlike that of any dinosaur yet known. Based on a high number of diagnostic differences between the skull of the Marathon specimen and other ankylosaurians, we consider it prudent to assign this specimen to a new genus and species of ankylosaurian. *Kunbarrasaurus ieversi* gen. et sp. nov. represents the second genus of ankylosaurian from Australia and is characterised by an unusual melange of both primitive and derived characters, shedding new light on the evolution of the ankylosaurian skull.

## Introduction

**Figure 1:**
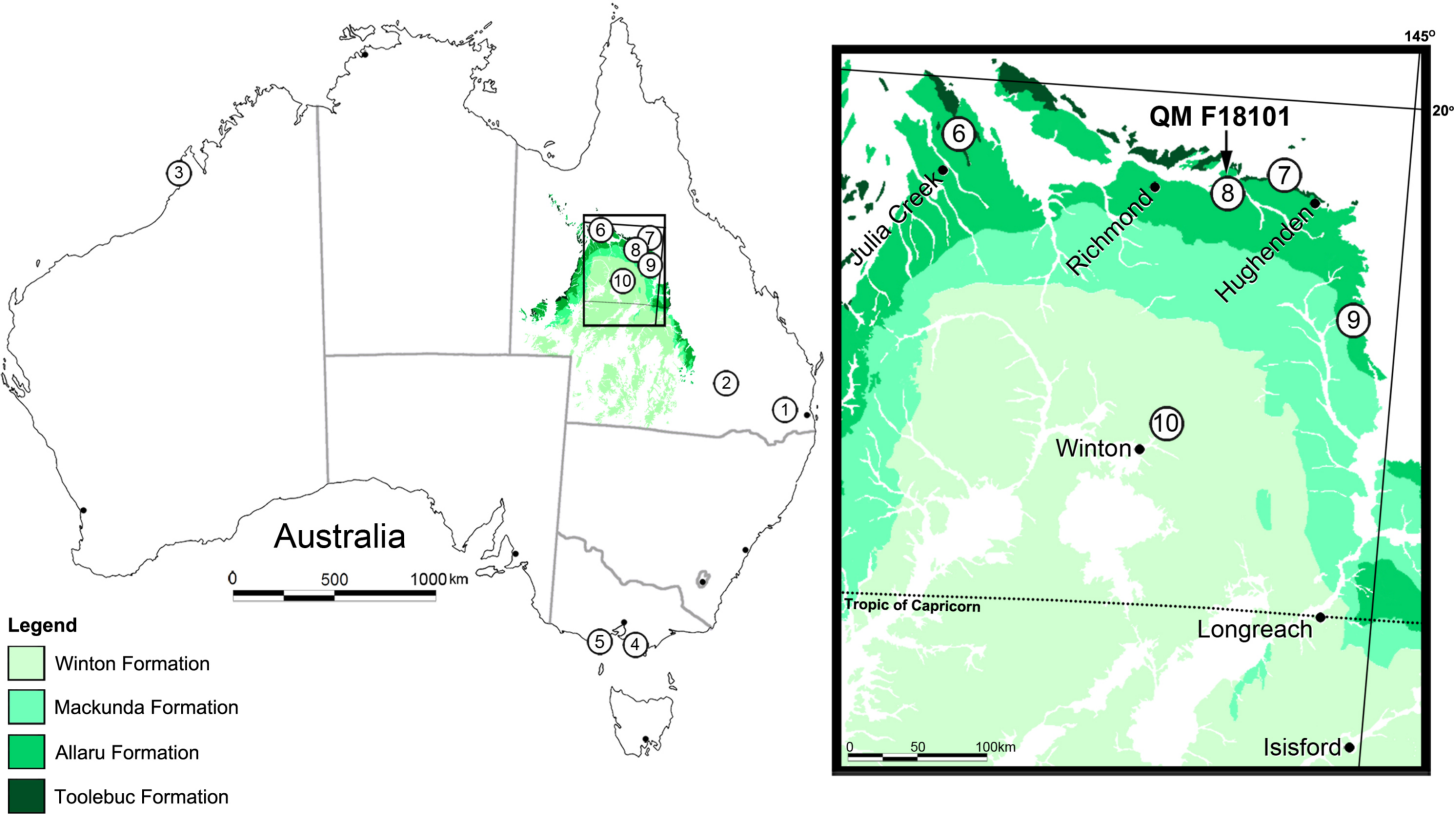
Locality map for Australian eurypodan thyreophoran fossils. 1, Stegosaurian? footprint (QM F5701), Walloon Coal Measures, Balgowan Colliery, Balgowan (Bajocian–Bathonian); 2, *Minmi paravertebra* holotype (QM F10329) (Molnar, 1980), Minmi Member, Bungil Formation (Valanginian–Barremian); 3, Thyreophoran trackways, Broome Sandstone, Dampier Peninsula, Western Australia (Valanginian–Barremian); 4, Ankylosauria indet. (see Barrett et al., 2010) ‘Flat Rocks’ Wonthaggi Formation (upper Hauterivian–Albian); 5, NMV P216739, ‘Lake Copco–Dinosaur Cove’ Eumeralla Formation (middle upper Aptian to lower middle Albian) (Barrett et al., 2010); 6, QM F33286; 7, AM F119849 and AM F35259; 8, *Kunbarrasaurus ieversi* gen. et sp. nov. (formerly *Minmi* sp.) (QM F18101); 9, QM F33565 and QM F33566; 10, QM F44324-28. Legend: Dark Green, Toolebuc Formation (late middle–early late Albian); Green, Allaru Formation (upper Albian–(?)lower Cenomanian); Light green, Mackunda Formation (upper Albian–lower Cenomanian); Lightest green, Winton Formation (late Albian–early Turonian).

In 1989 the near-complete skeleton of an armoured dinosaur (QM F18101) was found in the upper Albian–(?)lower Cenomanian Allaru Mudstone on Marathon Station near Richmond, north-western Queensland, Australia by Mr. Ian Ievers (Fig. 1). The fossil was collected and initial preparation was undertaken at the Queensland Museum. Recognising the scientific value of the specimen prior to preparation being completed, Molnar (1996a) published a partial description, and preliminarily assigned the specimen to *Minmi*—the only genus of ankylosaurian known from Australia. This assignment was based solely on its possession of paravertebral elements (ossified aponeuroses and tendons associated with the epaxial musculature), which were a diagnostic feature of *Minmi paravertebra* (Molnar, 1980). A complete description of the Marathon specimen and an assessment of its phylogenetic relationships, particularly its preliminary taxonomic assignment to *Minmi,* were planned for future research Molnar (1996a).

In total, seven ankylosaurian fossils from the Lower Cretaceous of Queensland have been attributed to *Minmi*, in most instances tentatively. Only two of these specimens have been described in any detail: the holotype *Minmi paravertebra* QM F10329 from the lower Aptian (Burger, 1980; Burger, 1993; Chan, 2009; Cook, Bryan & Draper, 2013) Minmi Member of the Bungil Formation near Roma (Molnar, 1980); and the Marathon specimen QM F18101 (preliminary assigned to *Minmi* sp. by Molnar, 1996a; discussed herein) (Fig. 1). The remaining five specimens were all discovered in the sediments of the north-western region of Queensland and include: QM F33286 from the upper middle–lower upper Albian (Gray, McKillop & McKellar, 2002; Cook, Bryan & Draper, 2013) Toolebuc Formation of Julia Creek; AM F119849 and AM F35259 from the Toolebuc Formation near Hughenden; and, QM F33565 and QM F33566 from the Allaru Mudstone near Hughenden (Fig. 1). Examination of these other specimens is currently being conducted as part of a broader study on Australian ankylosaurians.

In addition to the aforementioned specimens, fragmentary ankylosaurian material is also known from broadly coeval sediments in other parts of Queensland (Leahey & Salisbury, 2013) and Victoria (Barrett et al., 2010) (Fig. 1). Due to their isolated and/or fragmentary nature, taxonomic assignment of these specimens has not advanced beyond Ankylosauria indet.

Ichnological evidence of other thyreophorans is also known from Middle Jurassic (Bajocian–Bathonian) Walloon Coal Measures at Balgowan, Darling Downs, Queensland (Hill, Playford & Woods, 1966: 30–31; replica QM F5701) and the Lower Cretaceous (Valanginian–Barremian) Broome Sandstone of the Dampier Peninsula, Western Australia (Fig. 1) (Long, 1990; Long, 1992; Long, 1998; Long, 2002; Molnar, 1991; Thulborn, Hamley & Foulkes, 1994; Thulborn, 1998; Rich & Vickers-Rich, 2003; Scanlon, 2006; McCrea et al., 2012).

The Marathon specimen (QM F18101) represents the most complete dinosaurian fossil from eastern Gondwana (Australia, New Zealand, India and Antarctica), and the most complete ankylosaurian fossil from Gondwana. It is also one of the world’s most complete ankylosaurians. Moreover, it includes one of the few ankylosaurian skulls in which most of the sutures have not fused or been obliterated by dermal ossifications or remodeling of bone (Maryańska, 1971; Maryańska, 1977; Godefroit et al., 1999; Carpenter et al., 2001; Hill, Witmer & Norell, 2003; Burns et al., 2011).

Full preparation of the skull of QM F18101 has now been completed. In light of this advance, the aim of this paper is to provide a comprehensive description of the cranial osteology of QM F18101, in particular that pertaining to the nasal and palatal region. To this end, Computer Tomographic (CT) scanning, as well as digital segmentation and 3D computer visualisation of the endosseous cavities has also been undertaken. The results additionally enable us to compare QM F18101 with other ankylosaurian crania that have undergone similar analysis. The new description also allows for the taxonomic reassessment of QM F18101.

### Ankylosauria

Ankylosauria is a clade of quadrupedal, herbivorous ornithischian dinosaurs, whose members are characterized, in part, by extensive parasagittal rows of dermal ossifications on the dorsal and lateral surfaces of the neck, trunk and tail, and an unusual cranial architecture that incorporates ornamentation (also the result of dermal ossifications) (Vickaryous, Maryańska & Weishampel, 2004). Ankylosauria and its sister taxon Stegosauria are united to form the clade Eurypoda. Eurypodans and a number of closely related ornithischians that are similarly characterized by extensive dermal ossifications (e.g., *Scutellosaurus lawleri, Emausaurus ernsti* and *Scelidosaurus harrisonii*) collectively form the clade Thyreophora (Sereno, 1986; Sereno, 1999; Parish, 2005; Thompson et al., 2012). Ankylosauria generally includes two clades: Nodosauridae and Ankylosauridae (Sereno, 1986; Sereno, 1999; Coombs & Maryańska, 1990; Lee, 1996; Kirkland, 1998; Xu, Wang & You, 2001; Vickaryous et al., 2001; Vickaryous, Maryańska & Weishampel, 2004; Hill, Witmer & Norell, 2003; Thompson et al., 2012). However, a number of analyses also weakly support the inclusion of a third clade: Polacanthidae/Polacanthinae (see Kirkland, 1998; Carpenter, 2001; Parish, 2005).

Phylogenetic analyses that include *Minmi* (excluding those conducted by Leahey, Molnar & Salisbury, 2008; Leahey, Molnar & Salisbury, 2010; Arbour & Currie, in press) have treated the holotype *Minmi paravertebra* (QM F10329) and the Marathon specimen (QM F18101) as the same genus, despite the fact that the latter was only tentatively assigned to *Minmi* based on one defining feature (discussed above) and was not fully described or taxonomically assessed. Despite this, the specific phylogenetic position of *Minmi* has varied between analyses. This is most likely due to the limited published information on QM F18101’s osteology, both for the skull and the postcranium, as well as the fragmentary condition of *Minmi paravertebra*. Generally, *Minmi* has been assigned to two relatively basal positions within Ankylosauria. The taxon has been regarded as a stem ankylosaurian—that is positioned basal to the two major ankylosaurian clades (Kirkland, 1998; Carpenter, 2001; Leahey, Molnar & Salisbury, 2008; Leahey, Molnar & Salisbury, 2010; Arbour & Currie, in press). Other phylogenetic analyses have found *Minmi* to be a basal member of Ankylosauridae (e.g., Sereno, 1999; Xu, Wang & You, 2001; Hill, Witmer & Norell, 2003; Vickaryous, Maryańska & Weishampel, 2004; Thompson et al., 2012). Herein, we provide further information for the skull that may enable future clarification of the taxonomic relationships of QM F18101.

Regardless of the conflicting placements of QM F18101, its basal position in relation to Ankylosauria fits well with its ‘mid’ Cretaceous age. The early evolution of ankylosaurian thyreophorans has been difficult to decipher due to the rarity and fragmentary nature of specimens from the Jurassic and Lower Cretaceous, particularly in the landmasses that once comprised Gondwana. Due to its completeness and age, QM F18101 thus represents an ideal specimen with which to investigate aspects of the early evolution of Ankylosauria.

## Material and Methods

QM F18101 is housed in the Geoscience Collection of the QM. This study provides an analysis of the cranial osteology only. Cranial measurements are listed in Appendix S1.

This paper is an extension of previous work by Molnar (1996a) and Molnar (2001b) and includes descriptions of the previously unexposed cranial elements and ossifications as well as details revealed from CT imagery. To avoid repetition with Molnar’s previous work, the authors herein strongly advise the use of these earlier publications in conjunction with this description when utilising the information on QM F18101.

The skull of QM F18101 was CT scanned at the Mater Adult Hospital, South Brisbane, in accordance with the procedure outlined in Ridgely & Witmer (2006). The raw scan data were then used to reconstruct the endosseous cavities of the specimen as is outlined in Witmer & Ridgely (2008). The course of the airway will be described in relation to the habitual posture of the head, as has been done for other ankylosaurians described by Witmer & Ridgely (2008). Furthermore, all sutural boundaries were confirmed using the CT imagery utilising OsiriX (Vol 5.5).

The anatomical nomenclature follows the form as codified in the NAV (1994) and NAA (1993) as well as standardised terms used in dinosaur research (e.g., Coombs, 1978a; Witmer, 1995; Witmer, 1997; Vickaryous, Maryańska & Weishampel, 2004). The nomenclature for describing the endosseous cavities follows that outlined in Witmer & Ridgely (2008). The taphonomy of QM F18101 is discussed to some extent in Molnar (1996b), however a more detailed account will be published elsewhere alongside a detailed description of the entire postcranial skeleton.

The electronic version of this article in Portable Document Format (PDF) will represent a published work according to the International Commission on Zoological Nomenclature (ICZN), and hence the new names contained in the electronic version are effectively published under that Code from the electronic edition alone. This published work and the nomenclatural acts it contains have been registered in ZooBank, the online registration system for the ICZN. The ZooBank LSIDs (Life Science Identifiers) can be resolved and the associated information viewed through any standard web browser by appending the LSID to the prefix http://zoobank.org/. The LSID for this publication is: (urn:lsid:zoobank.org:pub:6B5B8495-206C-4341-B728-6F77A0A1BA7C). The online version of this work is archived and available from the following digital repositories: PeerJ, PubMed Central and CLOCKSS.

### Ontogenetic stage of the specimen

Our understanding of ontogeny in ankylosaurian dinosaurs is dominated by changes that occur in the postcranial skeleton (e.g., Maryańska, 1977; Coombs, 1986; Coombs, 1995; Coombs & Maryańska, 1990; Jacobs et al., 1994; Pereda Suberbiola, 1994; Godefroit et al., 1999; Pereda Suberbiola & Barrett, 1999; Xu, Wang & You, 2001; Hill, Witmer & Norell, 2003; Vickaryous, Maryańska & Weishampel, 2004), with more recent studies having utilised the microstructure of bone and various dermal ossifications (Hayashi et al., 2010; Stein, Hayashi & Sander, 2013; Ősi et al., 2014). Far-fewer ontogenetic changes have been recognised in the cranium. The most widely accepted change is the progressive fusion of the cranial sutures on the dorsal surface of the skull (e.g., Maryańska, 1977; Jacobs et al., 1994; Godefroit et al., 1999; Xu, Wang & You, 2001; Hill, Witmer & Norell, 2003; Vickaryous, Maryańska & Weishampel, 2004; Burns et al., 2011). Beyond this, most of the discussion on the identification of ontogenetic stages in ankylosaurians based on cranial morphology has been sourced from the numerous juvenile specimens of *Pinacosaurus* (Maryańska, 1971; Maryańska, 1977; Godefroit et al., 1999; Hill, Witmer & Norell, 2003; Burns et al., 2011) and other genera where numerous specimens are known (e.g., *Euoplocephalus* Penkalski, 2001; *Adontosaurus* Arbour & Currie, 2013). In all cases, however, it is unclear whether the traits discussed are truly ontogenetic or if they are the result of taxonomic differences or varying states of preservation Hill, Witmer & Norell, 2003; Penkalski, 2001; Arbour & Currie, 2013; Burns et al., 2011. For example, Burns et al. (2011) recently stated that larger specimens of *Pinacosaurus* have a greater number of marginal denticles on their teeth than smaller specimens of the same genus, thus this may represent an ontogenetic trait. However a reduced number of marginal denticles is also a feature of some nodosaurids (Coombs & Maryańska, 1990), thus it is possible that the feature Burns et al. (2011) suggested may be genus-specific ontogenetic trait, but clearly more specimens of other ankylosaurians and further investigation is necessary. Histological studies alongside gross morphological studies are likely to help assess whether these traits discussed by the aforementioned authors are ontogenetic or otherwise.

QM F18101 could be considered a sub-adult ankylosaur based on its small size along with the absence of fusion of the cranial elements, scapula-coracoid and pelvic elements (Molnar, 1996a), however Molnar argued that other factors suggest that it was near-mature to mature at death.

The lack of fusion of various cranial and postcranial elements seen in QM F18101 may not necessarily be related to immaturity, as these features are also plesiomorphic for the clade and cranial fusion may have developed only in more derived taxa (Molnar, 1996a). Two of the four known ankylosaurian skulls that exhibit cranial sutures are agreed to be juvenile specimens of the derived genus *Pinacosaurus*, a derived genus of Late Cretaceous ankylosaurian. However the other two, QM F18101 and *Cedarpelta* (Carpenter et al., 2001), are thought to be sub-adult–adult(?) and adult respectively. These taxa are positioned basally in relation to Ankylosauria, either as stem ankylosaurians (Leahey, Molnar & Salisbury, 2008; Leahey, Molnar & Salisbury, 2010; QM F18101 only, Arbour & Currie, in press) or basal members of clades within Ankylosauria (Hill, Witmer & Norell, 2003; Vickaryous, Maryańska & Weishampel, 2004; Thompson et al., 2012; *Cedarpelta*, only Arbour & Currie, in press). Furthermore, it is also noteworthy that a lack of fusion between the scapula and coracoid is also seen in some adult ankylosaurs (Coombs & Maryańska, 1990; Carpenter et al., 1999; Pereda Suberbiola, 1994), such that the ontogenetic utility of this feature may be more complex than previously thought.

Molnar (1996a) considered it noteworthy that all the specimens assigned to ‘*Minmi*’ (QM F10329, QM F33286, QM F33565, QM F33566, AM F35259, AM F119849) are approximately the same size (approximately 2.5–3 m total length) despite the fact that they originate from different locales and time periods, with the implication being that it would be highly unlikely that only juveniles were preserved. Furthermore the majority of early forms of ankylosaurs are small (less than 3 m e.g., *Mymoorapelta maysi* Kirkland & Carpenter, 1994, *Gargoyleosaurus parkpinorum* Kilbourne & Carpenter, 2005), compared with the more derived taxa from the Late Cretaceous of Asia and North America (approximately 6 m e.g., *Ankylosaurus* Carpenter, 2004). This would also suggest that the features that are typically considered ontogenetic in the more derived ankylosaurians may actually be the plesiomorphic condition.

A bone microstructure analysis was planned for this study in an effort to further assess the maturity of the specimen. Unfortunately preliminary testing of both *Minmi paravertebra* and QM 18101 revealed that the bone has undergone considerable remineralisation and oxidisation (a common process in surface rocks of these locales), such that histological investigations were not possible. A more detailed study of the ontogenetic status of QM F18101 is currently being undertaken with the description of its postcranial elements. For the present study, we concur with Molnar (1996a) that, in the absence of any unambiguous ontogenetic characters, it is most prudent to consider QM F18101 an almost mature or newly mature individual.

## Systematic Paleontology

**Table:** 

Dinosauria Owen, 1842
Ornithischia Seeley, 1888
Thyreophora Nopcsa, 1915
Eurypoda Sereno, 1986
Ankylosauria Osborn, 1923

*Kunbarrasaurus ieversi* gen. et sp. nov.

(formerly *Minmi* sp. Molnar, 1996a)

*Etymology.* The generic name combines *Kunbarra* [kunbara], the Mayi (Wunumara) word for ‘shield’, and *souros * (*σαυρoς*), the Greek word for ‘lizard’, and is a reference to the animal’s heavily ossified skin. The species name honours Mr Ian Ievers, discoverer of the holotype. The name therefore means ‘Ievers’ shield-lizard’.

*Holotype.* QM F18101, a near-complete ankylosaurian dinosaur specimen that includes most of the skull and mandible, along with an articulated postcranium.

*Diagnosis (cranial only).* The cranial roof is flat (excluding some very slight arching of the postorbital and nasal); the dorsal surfaces of the prefrontal, supraorbital and postorbital elements form an approximately 90° angle with the lateral surfaces of the skull; the supraorbital comprises a single element; the prefrontal is restricted to the dorsal surface of the skull and does not contribute to the orbit; the nasals do not contribute to the lateral surface of the skull, being restricted to the dorsal surface and the medially displaced nasal vestibule; the nasal vestibule (which is completely formed by the nasal bone) is large in proportion to the maxillary rostrum, fully exposed dorsally and laterally; the maxilla extends for the entire dorsoventral height of the skull and contacts the cranial roof at the prefrontal; the caudal end of the tooth row terminates ventral to the caudal margin of the orbit; the lacrimal is vertically (dorsoventrally) orientated; the pterygoids do not contact one another caudally near the braincase and are completely separated by the basisphenoid; the long axis of the quadrate is vertically aligned; the coronoid process is very pronounced; the sidewall of the braincase is poorly ossified, such that many of the cranial nerves must have traversed large bony apertures rather than the discrete foramina; the inner ear is extremely large in proportion to the skull and morphologically unlike any other known dinosaur in that the vestibular region is not separated by bone from the endocranial cavity, the ventral cochlear region is poorly ossified, and the vestibular region is so expanded that the semicircular canals appear very short; cranial dermal ossifications are either flat or with a very shallow keel; no quadratojugal and squamosal horn or boss-like dermal ossifications are present.

*Locality and horizon.* Marathon Station (south of the Flinders River) east of Richmond, north-western Queensland, Australia; Allaru Mudstone, uppermost part of the Wilgunya Subgroup of the Rolling Downs Group of the Eromanga Basin.

The Allaru Mudstone conformably overlies either the Toolebuc or the Wallumbilla formations, and is conformably overlain by the Mackunda Formation (Gray, McKillop & McKellar, 2002) (Fig. 1). The Allaru Mudstone is only exposed at the surface in an arch around western Queensland, but extends, subsurface, into the Northern Territory, South Australia and New South Wales. The unit comprises blue–grey, partly pyritic mudstones, interbedded with calcareous siltstones, cone-in-cone limestone and lesser, fine-grained sandstones. The latter are interbedded in the upper-most parts of the unit, and are laminated and thinly bedded, with labile lithic and calcareous components and carbonaceous partings (Gray, McKillop & McKellar, 2002) (Fig. 1).

Marine phytoplankton dominance fluctuates throughout the Allaru Mudstone, reflecting varying depositional environments. Numerous well-preserved marine macrofossils occur within it, including bivalves, gastropods and belemnites (Molnar, 1996a). Teleost fishes (e.g., Bartholomai, 2010; Bartholomai, 2012) and marine reptiles (*Platypterygius longmani* Wade, 1990; Elasmosauridae indet. Kear, 2003; *?Notochelone costata* Long, 1998) are also present. The combination of marine flora and fauna, with the largely mudstone-dominated lithology suggests that the Allaru Mudstone was deposited in low energy, shallow marine environments.

Despite the likely shallow marine depositional setting, a number of non-marine tetrapod fossils have also been discovered within the Allaru Mudstone. These include specimens attributable to pterosaurs (Molnar & Thulborn, 2007; Fletcher & Salisbury, 2010), the styracosternan ornithopod *Muttaburrasaurus* sp. (Molnar, 1996b; Agnolin et al., 2010), the non-titanosaurian somphospondyli sauropod *Austrosaurus mckillopi* (Longman, 1933; Coombs & Molnar, 1981; Molnar, 2001a; Molnar, 2011; Molnar & Salisbury, 2005; Agnolin et al., 2010; Poropat et al., 2015) as well as other ankylosaurian specimens provisionally assigned to *Minmi* (QM F33565 and QM F33566; Molnar, 1996a).

*Stratigraphic age.* Upper Albian–(?)lower Cenomanian (Gray, McKillop & McKellar, 2002), based mainly on the recognition of palynofloras indicative of the *Phimopollenites pannosus* spore-pollen zone (upper Albian: Dettmann & Playford, 1969; Helby, Morgan & Partridge, 1987; Gray, McKillop & McKellar, 2002; Cook, Bryan & Draper, 2013). In some regions the upper limits of the Allaru Mudstone incorporate the basal part of the *Hoegisporis uniforma* (formerly the *Appendicisporites distocarinatus*) spore-pollen zone (Cenomanian: Helby, Morgan & Partridge, 1987; Dettmann et al., 1992; Partridge, 2006), while some basal sections may extend into the upper part of the *Coptospora paradoxa* spore-pollen zone (lower Albian: Dettmann & Playford, 1969; Helby, Morgan & Partridge, 1987; Gray, McKillop & McKellar, 2002). A new study (Tucker et al., 2013), using U-Pb isotope dating of detrital zircons by laser ablation for selected strata in the Rolling Downs Group, suggests that some of the palynomorph zones may need to be temporally recalibrated, with the deposition of the Allaru Mudstone restricted to the upper Albian. This proposal would be consistent with the initial age suggested by Helby, Morgan & Partridge (1987). However, pending further work on this issue, we chose to consider the more conservative upper Albian–(?)lower Cenomanian depositional range for the unit.

## Description

### Preservation

The skull of QM F18101 has an unusual morphology for an ankylosaurian, and its state of preservation makes the interpretation of some aspects of its osteology problematic, particularly on the palate. For these reasons we have included a separate preservation section (a more detailed version will be available in Leahey, PhD thesis).

**Figure 2:**
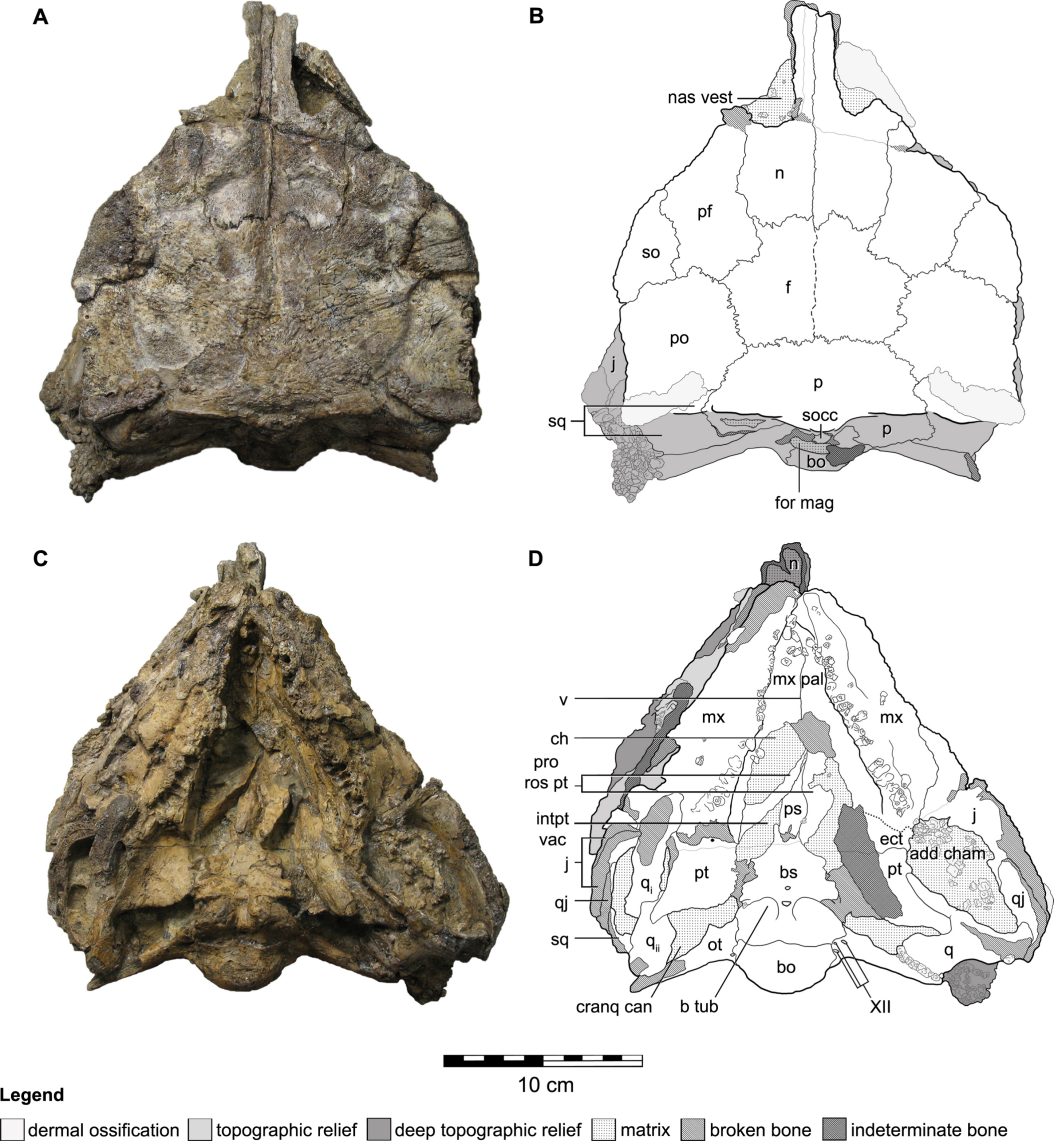
Cranial osteology of *Kunbarrasaurus ieversi* gen. et sp. nov. (formerly *Minmi* sp.) (QM F18101), with schematic version in dorsal (A–B) and ventral (C–D) aspects. Abbreviations: add cham, adductor chamber; bo, basioccipital; bs, basisphenoid; cranq can, cranioquadrate canal; ch, choanae; ect, ectopterygoid; f, frontal; fen pal, palatal fenestra; for mag, foramen magnum; intpt vac, interpterygoidal vacuity; j, jugal; mx, maxilla; mx pal, maxillary palate; n, nasal; nas vest, nasal vestibule; ot, otoccipital; p, parietal; pf, prefrontal; po, postorbital; pro rost pt, rostral process of the pterygoid; ps, parasphenoid; pt, pterygoid; q, quadrate (parts i and ii); qj, quadratojugal; so, supraorbital; socc, supraoccipital; sq, squamosal; v, vomer; XII, hypoglossal nerve. Legend: dotted line, partially fused suture; light grey, dermal ossifications; grey, topographic relief; dark grey, deep topographic relief; slanted lines, broken surfaces; dots, matrix; hatching, fragmentary bone material. Scale bar equals 10 cm.

**Figure 3:**
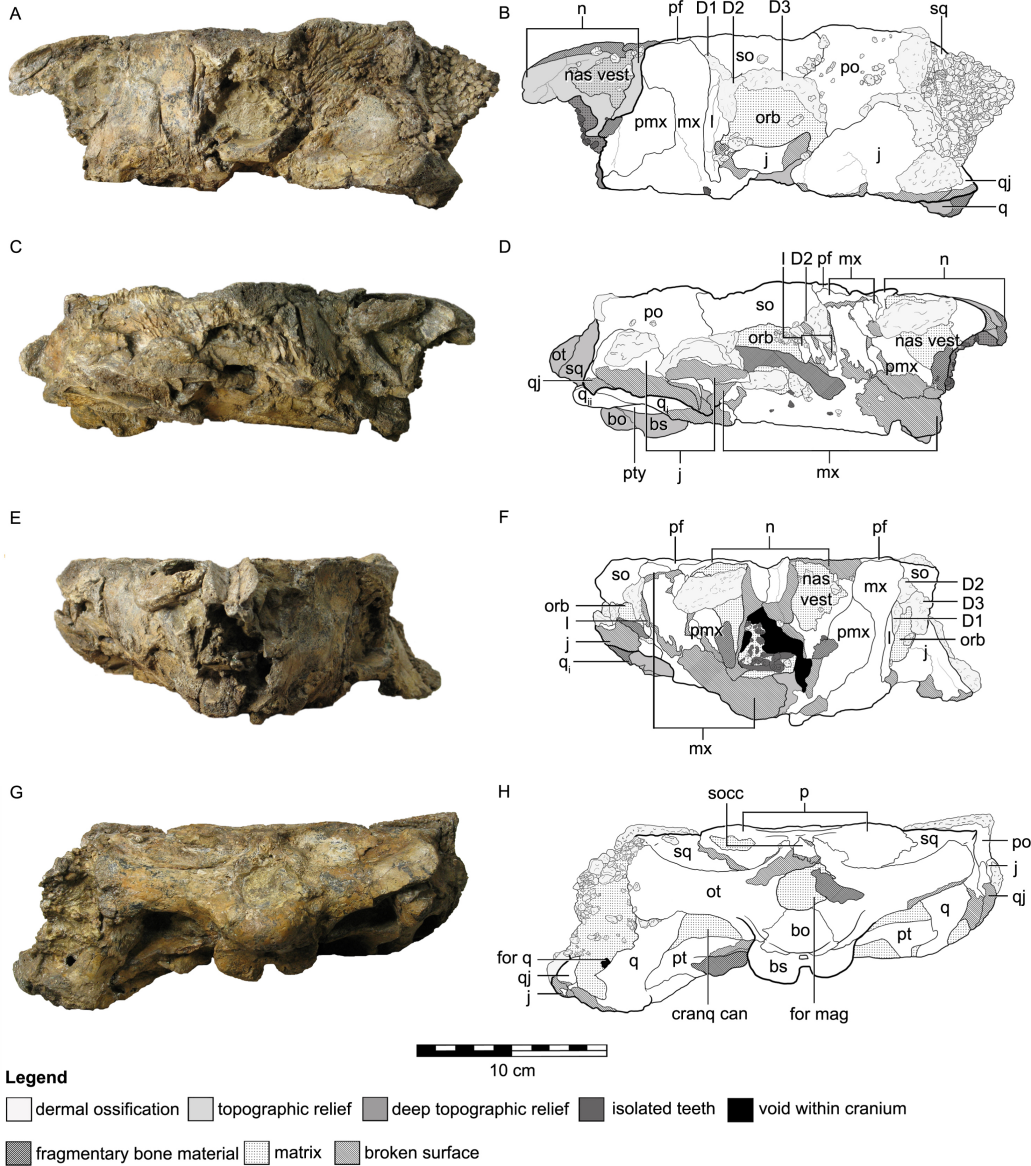
Cranial osteology of *Kunbarrasaurus ieversi* gen. et sp. nov. (formerly *Minmi* sp.) (QM F18101), with schematic version in left lateral (A–B), right lateral (C–D), rostral (E–F) and caudal (G–H) aspects. Abbreviations: bo, basioccipital; bs, basisphenoid; cranq can, cranioquadrate canal; D, dermal ossification; for q, quadrate foramen; for mag, foramen magnum; j, jugal; l, lacrimal; mx, maxilla; n, nasal; nas vest, nasal vestibule; orb, orbit; ot, otoccipital; p, parietal; pf, prefrontal; pmx, premaxilla; po, postorbital; pt, pterygoid; q, quadrate (parts i and ii); qj, quadratojugal; so, supraorbital; socc, supraoccipital; sq, squamosal. Legend: dotted line, partially fused suture; light grey, dermal ossifications; grey, topographic relief; dark grey, deep topographic relief; darkest grey, teeth (loose); black, void within skull; slanted lines, broken surfaces; dots, matrix; hatching, fragmentary bone material.

The skull of QM F18101 is approximately 90 per cent complete (Figs. 2 and 3). The majority of sutures are evident; only the suture between the parietals is completely fused, while the suture between the frontals is partially fused but still discernable. Matrix and dermal ossifications (mainly ossicles) occupy the nasal vestibules, orbits and adductor chambers. Matrix, ossicles and isolated teeth obscure parts of the rostral opening of the nasal cavity and the choanae. Matrix covers the dorsal two-thirds of the braincase and is also present in the interptyergoid vacuity, cranioquadrate canal and the foramen magnum. Numerous fractures transect the skull, and some of these have additionally resulted in the displacement of some elements (see Figs. 2 and 3).

**Figure 4:**
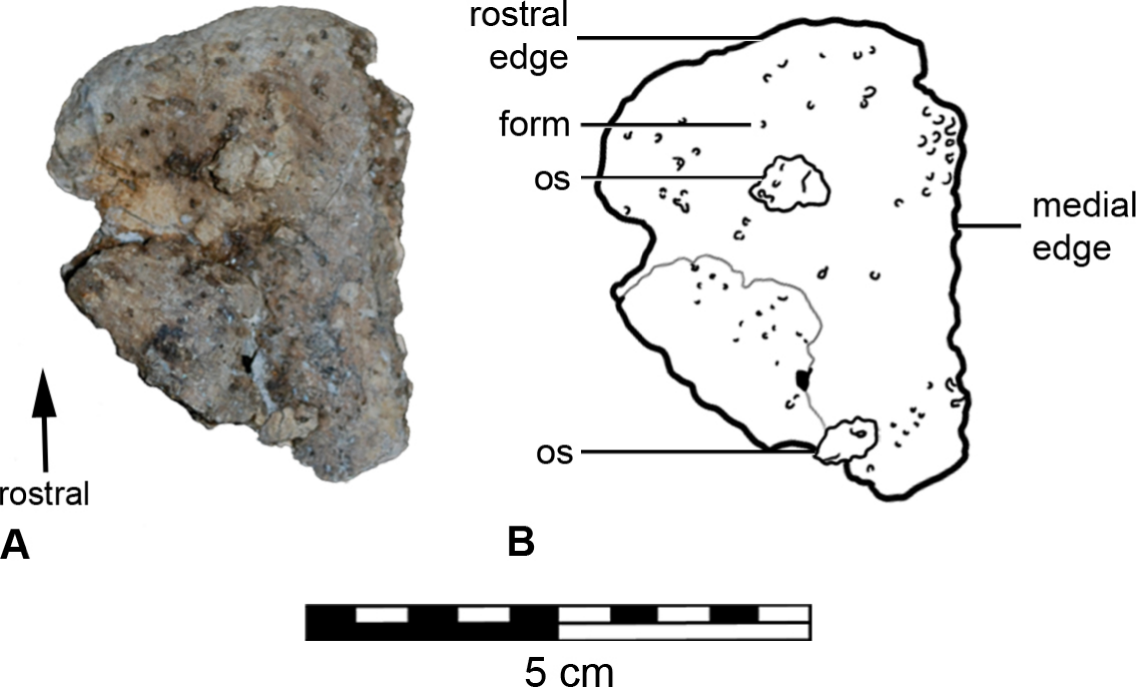
Left fragment of the premaxillary beak (A) with schematic (B), in rostral aspect.

The apex of the maxillary rostrum is not preserved, such that the nares as well as the rostral-most parts of the premaxillae, maxillae and nasals are missing. The left premaxillary ‘beak’ was removed from the nasal vestibule during preparation of the skull (see Molnar, 2001b: Fig. 16.5, N.B. it is not labelled) (Fig. 4). The right premaxillary beak was not recovered.

The left lateral side of the skull is well preserved (Figs. 3A and 3B), but some slight dorsoventral crushing has occurred to the ventral edge of the maxilla and the lacrimal. The orbital process of the jugal has broken and has been displaced slightly dorsally, its rostral tip is lost. The ventral margin of the jugal and quadratojugal are not preserved. Osteoderms, ossicles and matrix obscure (from left lateral aspect) the caudal-most edge of the left lateral side of the skull (parts of the squamosal, postorbital, jugal, quadratojugal, quadrate, and the quadrate foramen) (Figs. 3A and 3B).

The right lateral side of the skull has been crushed ventromedially (Figs. 3C and 3D), affecting the preservation of all elements from the right lateral side. Surface bone is missing in parts, some elements are highly fragmented and all bones have moved from their original positions: the right nasal vestibule is laterally flattened; the ventral part of the right maxilla, including the maxillary tooth row, has twisted medially and now sits within the oral cavity obscuring parts of the palate; the ventral parts of the right jugal and quadratojugal have broken and now lie lateral to the cheek region; the orbital process of the jugal has also moved caudolaterally; and, the ventral-most part of the squamosal is missing. A long piece of bone of unknown origin extends across the orbital cavity (Figs. 3C and 3D).

In ventral aspect (Figs. 2C and 2D), a considerable amount of crushing is apparent, with the majority of the ventrally exposed bones exhibiting varying degrees of fragmentation. The right maxilla has rotated medially into the oral cavity, disarticulated caudally, and now overlies the rostral edge of the right pterygoid and the caudal end of its rostral process. The caudal-most part of the right maxilla and right ectopterygoid are missing. Although highly fragmented the left maxillary tooth row remains in its *in vivo* position. Extensive fracturing obscures the sutural boundaries of the left ectopterygoid. The roots of fourteen teeth remain in their alveoli in the right maxilla, and twenty in the left maxilla. Some disarticulated and broken crowns and roots are scattered on the maxillae. The maxillary secondary palate and the vomer exhibit fragmentation as a consequence of the medially turned right maxillae. The caudal edge of the maxillary secondary palate and the vomer are not preserved (Figs. 2C and 2D). The location, or presence, of the palatines is not clear. Additionally the resolution of the CT imagery is poor in this area and thus they cannot be located via this method (Figs. 2C and 2D). In ventral aspect, the parasphenoid has disarticulated from the basisphenoid, and the adjacent areas are slightly damaged. The rostral processes of the pterygoids have moved laterally slightly relative to the parasphenoid. Collectively, the palatal surfaces of the parasphenoid and the rostral processes of the pterygoids are directed slightly dorsal and to the left lateral (Figs. 2C and 2D). The rostral process of the left pterygoid has disarticulated and rotated medially, such that its lateral surface now lies in the horizontal plane. An irregular-shaped portion of the lateral surface of the left pterygoid is missing. The caudal ends of the rostral processes of both pterygoids are obscured by matrix. The left pterygoid remains in natural articulation, apart from its contact with the basisphenoid. Lateral to this articulation, the medial half of the ventral surface of the left pterygoid is missing. A fragment of the bone, possibly from the mandible, overlies most of the ventral surface of the left pterygoid. The right pterygoid-basisphenoid contact is also broken, and the right pterygoid now lies slightly dorsolateral to the basisphenoid, such that it is unclear if the pterygoid–basisphenoid suture was fused. A small portion of the mandibular process of the right pterygoid is broken (Figs. 2C and 2D). The ventral edges of the jugals, quadrates (including the articular condyles) and quadratojugals are not preserved. The right quadrate is displaced, a fracture across its shaft, so that it now lies in the horizontal plane, across the adductor chamber. The mandibular condyle of the right quadrate is now angled rostrally and overlies the caudal end of the right maxilla, lateral to the caudal-most alveolus (Fig. 2D ‘q_i_’), whereas the squamosal-articular end of the right quadrate underlies the right pterygoid but overlies the lateral end of the right otoccipital (Fig. 2D ‘q_ii_’).

Although the lateral walls of the braincase (Figs. 2C and 2D) have some minor fractures, the general regions that comprise it (e.g., the prootic, laterosphenoid, orbitosphenoid) are discernible in the CT scan data, as are some but not all of the sutures between them.

In caudal aspect, all elements are preserved with some minor fragmentation (see Figs. 3G and 3H), however much of the supraoccipital is missing, thus exposing the parietal–otoccipital–supraoccipital contact.

The dentary and the coronoid process of the left mandibular ramus are partially preserved, whereas only a portion of the dentary from the right mandibular ramus remains.

### Osteology

Overall, the skull is approximately 30 per cent longer than it is wide. In dorsal aspect, the skull has a pentagonal outline, with the greatest width occurring immediately caudal to the orbits. In caudal aspect the occiput is transversely wider than it is high dorsoventrally (Figs. 3G and 3H), much of it can also be observed in dorsal aspect (Figs. 2A and 2B). In ventral aspect the skull is triangular in outline, with the greatest width towards the caudal edge, and the tooth rows are medially inset (Figs. 2C and 2D). In lateral aspect, the cranial roof is flat, rising slightly dorsally immediately caudal to the orbits (i.e., at the postorbital). There is also a slight arching of the maxillary rostrum (within the rostral half of the nasals) (Figs. 3A–3D). The dorsal surfaces of the prefrontals, supraorbitals and postorbitals form an approximately 90° angle with the lateral surfaces of the skull. Dermal ossifications are present on the skull (see below and Molnar, 2001b). The majority of cranial sutures on QM F18101 are unfused, with the exception of some of those involved in the braincase. Other sutures may be obscured from view by dermal ossifications (Figs. 2 and 3). The antorbital, supratemporal and lateral temporal fenestrae are closed but a quadrate foramen is retained (Figs. 2A–2B and 3A–3B).

### Rostral region

#### Premaxilla

**Figure 5:**
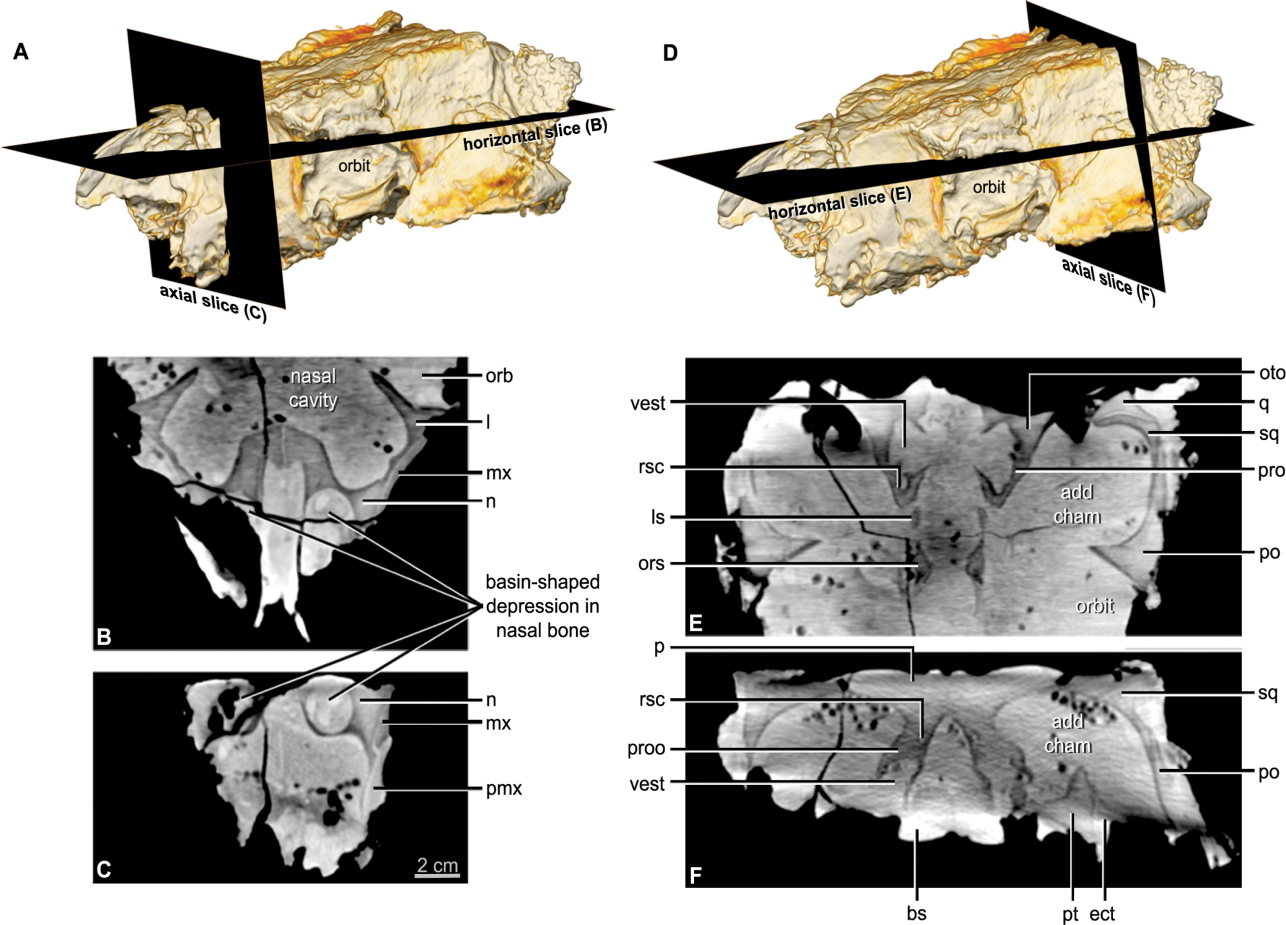
CT scan slices through the nasal cavity A, B, C, and braincase D, E, F regions of *Kunbarrasaurus ieversi* gen. et sp. nov. (formerly *Minmi* sp.) (QM F18101). (A, D) Volume rendering of the entire skull showing the positions of the horizontal slices in (B, E) and the axial (vertical) slice in (C, F). Abbreviations: ls, laterosphenoid; ors, orbitospenoid; oto, otoccipital; proo, prootic; rsc, rostral semicircular canal; vest, vestibule. For other abbreviations see Fig. 1. Scale bar equals 2 cm.

The premaxilla comprises a main lateral body and a rostral beak. The main body of the premaxilla forms approximately 25% of the total length of the preserved portion of the skull in lateral aspect (Figs. 3A–3D). The premaxilla is a vertically orientated plate. The caudal-most third is rectangular and completely bordered by the maxilla. Immediately rostral to this, the dorsal margin sharply rises dorsally at approximately 70°, to a point where the sutures of the nasal, premaxilla and maxilla intersect. Rostral to this point the dorsal margin of the premaxilla extends rostroventrally towards its ventral margin at approximately 45°. The dorsal margin of the premaxilla is bordered dorsally and medially by the nasal. The dorsal edge of the premaxilla and the lateral-most edge of the nasal contact each other at an oblique angle. This contact occurs lateral to the nasal vestibule. It is unclear if the premaxilla contributes to the border of each naris. The premaxilla is bordered ventrally by the maxilla along its entire preserved length. The ventral margin, rostral to the caudal rectangular portion of the premaxilla (described above), dips sharply ventrally and then grades more gently towards the apex of the maxillary rostrum. Thus the rostral two-thirds of the premaxilla tapers towards the apex of the maxillary rostrum. The internal surface of the premaxilla is concave and contributes to the rostral ventrolateral walls of the nasal cavity. The interior surface and rostral-half of the exterior surface of the premaxilla consist of smooth bone (Fig. 5C). Along the ventral margin of the left premaxillae is an area that is roughened with scattered foramina (Fig. 3A). Such foramina are often cited as evidence for the possible presence of a rhamphotheca, particularly amongst ornithischian dinosaurs (Barrett, 2001), although Cuff & Rayfield (2015) noted that the presence of premaxillary foramina alone is not sufficient evidence. Nevertheless, many workers have regarded a diversity of ankylosaurs as having beaks that likely were covered with a rhampotheca (Coombs, 1971; Coombs & Maryańska, 1990; Rybczynski & Vickaryous, 2001), an interpretation that we follow here for QM F18101.

The preserved portion of the left premaxillary beak (Fig. 4) has a triangular outline approximately 50 mm dorsoventrally long and 40 mm at its greatest width. The medial and lateral edges are straight. The rostral edge curves at its junctions with the medial and lateral edges, thus suggesting the presence of a premaxillary notch. The element is concave internally and very slightly convex externally, the rostral edge curves at a greater angle. It is at the most 3 mm thick on the medial edge, and thins towards the lateral. The external surface is dotted by foramina, some considerably deep, and the internal surface is smooth.

#### Maxilla

The maxilla contributes approximately one-third of the total length of the preserved portion of the skull in lateral aspect (Figs. 3A–3D), and two-thirds in ventral aspect (Figs. 2C and 2D). In lateral aspect the maxilla is bordered by the premaxilla rostrally, the nasal rostrally and dorsally, the prefrontal dorsally, the supraorbital dorsocaudally, and the lacrimal and jugal caudally. In lateral aspect, the maxilla extends for the entire dorsoventral height of the skull and contacts the cranial roof (at the prefrontal). In lateral and rostral aspects, it is unclear if the maxillae contribute to the external nares, but based on the maxilla’s slope (in lateral aspect) it is most likely that the maxillae do not contribute to the external narial borders. The tooth row, in lateral aspect, arches ventrally slightly for about half the tooth row length at approximately one-quarter of the way along its length from the rostral apex. Along the ventral margin of the left maxilla is an area that is roughened with scattered foramina scattered throughout it, and may suggest extension of the rhampotheca onto the maxilla or could reflect simply the passage of neurovascular canals to the oral margin.

In ventral aspect, the maxilla underlies the orbit and part of the jugal, thus the orbit is obscured from view. The pterygoid forms the caudal border to the maxilla. In ventral aspect, the tooth row is inset from the lateral edge of the maxilla. As the rostral portions of the lateral maxillary shelves (buccal emarginations) are slightly damaged, their exact width cannot be determined. Caudally, the shelf remains wide as in *Pinacosaurus* (AMNH 6523), *Ankylosaurus* (AMNH 5214), and *Euoplocephalus* (AMNH 5405), whereas it becomes narrow in *Saichania* (PIN N 3142/250). The tooth row shows a slight hour-glass shape, in ventral aspect. The lateral divergence of the tooth rows at their caudal ends is considerably wider than the rostral end. The caudal end of the tooth row is unusual in that it is below the caudal margin of the orbit. More typically the tooth row ends well before the orbit (*Gastonia* CEUMP 1307, *Ankylosaurus* AMNH 5214, *Euoplocephalus* AMNH 5405, *Panoplosaurus* ROM 1215, *Edmontonia* USNM 11868, *Pawpawsaurus* SMU 73203), below the rostral margin of the orbit (*Pinacosaurus* AMNH 6523, *Saichania* MPC 100/151 and HBV 10001, *Gargoyleosaurus* DMNH 27726, *Silvisaurus* KUVP 10296), or below the orbit (*Saichania chulsanensis* PIN 551/29—previously *Tarchia* (Arbour & Currie, in press)). There are 25 roots or alveoli in the left maxilla and 23 in the right. This contrasts with 20 teeth in the right maxilla and 18 in the left in *Saichania* (Carpenter et al., 2001), 19 in *Maleevus* and *Tsagantegia* (Tumanova, 1993) and also *Nodocephalosaurus* (Sullivan, 1999), 17 in *Pinacosaurus* (Maryańska, 1977), 16–17 in *Tarchia kielanae* (Miles & Miles, 2009), 34–35 in *Ankylosaurus* (Carpenter, 2004), and 19–24 in *Euoplocephalus* (Vickaryous & Russell, 2003). Thus, although the tooth count is higher than is typical for ankylosaurians, it is not abnormally high as in *Ankylosaurus*. Rostrally, each tooth position (alveolus) of QM F18101 has at least one replacement alveolus, but this number increases caudally to as many as three.

Between the tooth rows, the medial extensions of the maxillae form the maxillary secondary palate. These medial extensions are separated by a thin, vertically orientated bone, most likely the vomer. The caudal edge of the maxillary secondary palate forms the rostral and lateral borders of the primary choanae (internal nares). This maxillary secondary palate extends from the rostral tip of the maxilla to a point just rostral of the orbits. The maxillary secondary palate forms a horizontally flat plate, and is the ventral surface of the nasal cavity.

#### Teeth

The dental morphology of this specimen is discussed in detail by Molnar (1996a). The maxillary teeth differ from those of other basal ankylosaurians, such as *Gastonia* (CEUMP 1307) and *Gargoyleosaurus* (DMNH 27726) in that the crowns of QM F18101 have a well-developed cingulum on both sides, although not as bulbous as that of some nodosaurids (*Edmontonia* USNM 11868, *Panoplosaurus* ROM 1215) or some ankylosaurids (*Saichania* MPC 100/151). The teeth are more nodosaurid-like than ankylosaurid-like in the reduced number of marginal denticles (QM F18101 has 7 denticles Molnar, 1996a).

#### Nasal

The nasal comprises over one-third of the skull in rostral aspect (Figs. 3E and 3F) and approximately one-quarter of the lateral and dorsal lengths of the skull (Figs. 2A–2B and 3A–3B). In lateral aspect the nasal is bordered dorsolaterally by the maxilla and ventrolaterally by the premaxilla. In dorsal and lateral aspects, the nasals arch slightly dorsally over the maxillary rostrum. The nasals do not contribute to the lateral surface of the skull, being restricted to the dorsal surface and forming the medially displaced nasal vestibule (discussed below). Contrary to Molnar (1996a) the position of the nares is not lateral to the maxillary rostrum/‘anterior moiety’ of the nasals, visible in rostral aspect (Molnar, 1996a: Fig. 10, labelled ‘R’). CT imagery revealed that these are blind-ending chambers. The nasal bones provide the rostral portion of the roof and part of the lateral wall of the nasal cavity (see below). Each nasal bone has a large ventrolateral swelling, which projects into the nasal cavity. The paired swellings contact each other caudally near the suture with the frontal bone, but they diverge from each other more rostrally. Each swelling is hollowed out rostrally such that it forms a rostrally-open basin-shaped cavity (Figs. 5B and 5C: labelled ‘R’ Molnar, 1996a: Fig. 10). This nasal basin is blind caudally except for a presumably vascular canal that passes caudally from the basin, extends through the nasal swelling, and opens more caudally in the lateral chamber of the nasal cavity. Vascular canals associated with the roof of the nasal cavity have been identified in other ankylosaurians (Witmer & Ridgely, 2008; Miyashita et al., 2011). The CT scan data reveals that there is no ossified nasal septum in QM F18101, but there is a median plane of radiodensity that is different from both the bone and matrix (Fig. 5C), suggesting that the cartilaginous nasal septum (which is ubiquitous in amniotes) may have been somewhat mineralized in QM F18101. In ankylosaurians the nasal septum variably mineralizes (e.g., *Edmontonia* AMNH 5381) and in some cases ossify (*Euoplocephalus* AMNH 5405).

#### Prefrontal

In dorsal aspect, the prefrontals are bordered medially by the nasals, caudomedially by the frontals, caudally by the postorbitals and caudolaterally by the supraorbitals (Figs. 2A and 2B). The edges of the prefrontals overlie the maxillae laterally and the lateral-most tip of the nasals. The prefrontal is restricted to the dorsal surface of the skull and does not contribute to the orbit. The condition found in non-eurypodan thyreophorans and other ankylosaurians is for the prefrontal to extend onto the lateral surface of the skull. The prefrontal in QM F18101 is similar to *Pinacosaurus* (Maryańska, 1977) in that it is excluded from the orbital margin by the supraorbital. In ankylosaurians and non-eurypodan thyreophorans the prefrontal contributes to the orbital margin (Vickaryous, Maryańska & Weishampel, 2004)

In cross section, each prefrontal has a triangular profile, which becomes larger caudally. In cross section, the caudal extremity of the bone becomes more angular in outline, forming an inverted ‘L’ shape. The ventral extension of the prefrontal forms part of the dorsal half of the orbit and part of the dorsolateral wall of the nasal cavity, thus separating the orbital and nasal cavities from one another.

#### Lacrimal

The lacrimal is a plate-like bone, vertically orientated in the sagittal plane. The rostral-most portion is situated medial to the maxilla and ventral to the supraorbital, and thus is not exposed. Caudally the plate becomes laterally exposed and extends over the maxilla, thereby forming the rostral edge of the orbit (Figs. 3A–3D). In lateral aspect, the exposed portion of the lacrimal forms a dorsoventrally thin wedge. Additionally, in cross-section, the lacrimal becomes thinner and more concave caudally, forming approximately half of the orbital margin rostroventrally. CT imagery reveals that the nasolacrimal duct is housed within the lacrimal. It opens laterally into the orbit just ventral to the prefrontal-lacrimal contact and rostrally into the nasal cavity, dorsal to the maxilla.

### Temporal region

#### Supraorbital

The supraorbital is sub-rectangular in cross section. Rostrally, the ventral surface is slightly concave, forming the dorsal wall of the orbit. Caudally, in cross section, the rostral and caudal edges of the bone thin, resulting in a fusiform shape. The form of the right supraorbital bone differs slightly to that of the left due to the fusion of some dermal ossifications (see ‘Dermal ossifications’) (Figs. 3A–3D).

There is only a single supraorbital on each side of the skull (Figs. 2A–2B and 3A–3D), as noted by Molnar (1996a), and there is no evidence of sutures within either element. All other thyreophorans (with visible cranial sutures) possess three supraorbital elements e.g., *Pinacosaurus* (ZPAL MgD-II/1; Maryańska, 1977), *Scelidosaurus harrisonii* and stegosaurians (Maidment & Porro, 2009). It is unclear if *Cedarpelta* possessed three elements, as no sutures are visible (Carpenter et al., 2001).

#### Postorbital

Molnar (1996a) originally described the postorbital as a composite bone with the squamosal and postfrontal, therein known as the ‘squamosal’. The squamosal and postorbital are herein described as separate bony elements (discussed below). Molnar’s (1996a) inclusion of a postfrontal in his ‘composite squamosal’ was based on comparisons with the juvenile *Pinacosaurus grangeri* (Maryańska, 1977); however, the ‘postfrontal’ of this specimen is now understood to be a supraorbital as the postfrontal is lost in all dinosaurs (Maidment & Porro, 2009). Thus it is most unlikely that QM F18101 possessed a postfrontal.

Close examination of Molnar’s (1996a) ‘squamosal’ (postorbital + squamosal) has revealed that these bones are actually separate (Figs. 2A–2B, 3A–3D and 3G–3H). CT imagery further reveals that the sutural contact between the two elements is continuous (Fig. 5F). The postorbital is restricted to the lateral and dorsal surfaces. The postorbital lies slightly dorsal and lateral to the squamosal. It forms the caudodorsal corner of the skull. The postorbital-squamosal contact runs laterally beneath the osteoderms, and emerges caudolaterally, immediately ventral to these osteoderms. Caudolaterally, the suture turns at 90° onto the lateral surface of the caudolateral edge of the skull. Due to overlying matrix and preservation, the full extent of the lateral surface contacts is unclear. Thus the postorbital, in dorsal aspect, has a laterally compressed hexagonal outline, and contacts the supraorbital rostrally, the prefrontal rostromedially, the frontal medially, the parietal caudomedially and the squamosal caudally (Figs. 2A and 2B). In lateral aspect the postorbital is squarish in outline, and contacts the supraorbital rostrally, the jugal ventrally and the squamosal caudally (Figs. 2A–2D). In both dorsal and lateral aspects, the postorbital forms almost a third of the entire lateral and dorsal edges of the skull, respectively.

In lateral aspect, the postorbital arches above the level of the dorsal margin of the rostral two-thirds of the skull (Figs. 2A–2D). The arch begins at the caudal quarter of the orbital margin and reaches its apex in line with the caudal margin of the orbit. This arch then grades ventrally towards the caudal margin of the skull, and levels at where the dorsal osteoderms are situated. The ventral two–thirds of the rostral border of the postorbital forms the caudodorsal quarter and two–thirds of the caudal border of the orbit. The ventral margin of the postorbital overlies the dorsal edge of the jugal, arching dorsally at approximately 45°.

CT imagery reveals that the postorbital extends a postorbital wall or lamina medially, partially closing off the orbit from the adductor chamber (Fig. 5E). Caudal to this the internal (ventral) surface of the postorbital is mostly concave and contributes to the dorsolateral wall of the adductor chamber. At approximately half the length of the chamber is a ventrally angled process, most likely for the attachment of the adductor muscles.

Molnar (1996a) also noted a network of semi-parallel open canals with foramina on the lateral surface of the postorbital (the ‘squamosal’ of Molnar, 1996a). Ornamentation on the postorbital is also known in stegosaurians and *Scelidosaurus* (Maidment et al., 2008; Maidment & Porro, 2009). The ‘vascularisation’ of QM F18101 is reminiscent of the vascular canals that occur on the bony plates of stegosaurians (Buffrenil, Farlow & De Ricqles, 1986).

#### Jugal

The jugal forms the lateral wall of the adductor chamber (Figs. 2A–2D). Its rostral-most portion is situated medial to the postorbital, but caudally it wraps around lateral to it. The caudal third of the bone, that which is ventral to the squamosal, flares laterally. It does not completely obscure the quadrate.

#### Frontal

The pair of frontals are joined along the midline, with the undulating suture still visible however partially fused (Figs. 2A and 2B). The suture is also visible in the CT imagery. As discussed previously, the frontals contact the postorbitals caudolaterally. In cross section the frontal is rostrally thin but thickens caudally. The rostral third of the ventral surface is slightly concave. This concavity increases caudally and is subdivided by a longitudinal swelling that partially partitions the nasal cavity in this region into a narrow medial region and a broader lateral region. This lateral region is bounded laterally by a ventral crest separating the nasal cavity and orbit that is continuous with the same crest on the prefrontal bone noted above. This crest is continuous caudally with the crista cranii of the frontal, which is the sharp crest separating the orbit from the endocranial cavity. Further caudally, the crista cranii contacts the orbitosphenoid to form the lateral wall of the olfactory tract of the cranial endocast (see below).

#### Parietal

The parietals are fused medially to form a single element (Figs. 2A, 2B and 5F). In dorsal aspect, the bone has a semicircular outline, with the straight edge forming the caudal margin of the dorsal surface of the skull. At this margin, the parietal curves ventrally at an approximately ninety-degree angle, extending onto the occiput. In dorsal aspect, the parietal contacts the frontals rostrally, the postorbitals rostrolaterally and the squamosals caudolaterally. In caudal aspect, it overlays the medial portions of the squamosals, but is overlain by the otoccipital and the supraoccipital medially (Figs. 3G and 3H). In transverse section, the ventral surface of the parietal is concave as it roofs the endocranial cavity, with the degree of concavity increasing caudally, consequently producing ventrally directed extensions laterally that contact the laterosphenoid, prootic, and otoccipital bones to form the side wall of the braincase (Fig. 5F).

#### Squamosal

Contrary to Molnar (1996a), the squamosal is an individual element, not part of a composite bone (discussed above in the description of the postorbital). The squamosal forms the lateral parts of the caudal margin of the skull and is visible in dorsal and caudal aspects (Figs. 2A–2B and 3G–3H). The squamosal sits slightly ventral to the postorbital. The lateral surface is angled slightly caudally, and is only just visible in lateral aspect. The caudal surface is also angled slightly dorsally, such that where the lateral and caudal surfaces meet there is a caudally angled apex. This occurs immediately rostral to the dorsolateral corner of the paroccipital process. In dorsal aspect, the squamosal has a rectangular outline, being longer mediolaterally than it is rostrocaudally, it contacts the parietal medially and the postorbital rostrally. In caudal aspect, the squamosal sits ventral to the parietal medially and the otoccipital caudally. The left squamosal appears to extend beneath the majority of the left parietal and CT imagery reveals that the squamosal extends ventrally about half the height of the paroccipital process (Figs. 5E and 5F). CT imagery also shows that the internal surface of the squamosal is concave, allowing it to accommodate the curve of the quadrate. Furthermore, CT imagery reveals that the squamosal contacts the quadratojugal ventrally in lateral aspect.

#### Quadratojugal

In ventral aspect, the outline of the quadratojugal is triangular, with the medial surface being curved (Figs. 2C and 2D). It is situated lateral to the quadrate and medial to the jugal, thus contributing to the caudolateral margin of the subtemporal fossa. CT imagery reveals that the quadratojugal contacts the squamosal dorsally in lateral aspect. This rostral, dorsally orientated extension forms the rostral margin of the quadrate foramen. Furthermore the dorsal margin of quadratojugal is dorsally concave forming the ventral margin of the quadrate foramen, adjacent to a smooth-surfaced notch on the quadrate completing the boundary for the quadrate foramen.

### Palatal region

#### Vomer

The vomer is a fused element. It is a thin, vertically orientated plate that is fused to the maxillary bony secondary palate (Figs. 2C and 2D). This portion of the bone contributes to the nasal septum, but it is unclear to what extent dorsally, because, as noted above (see ‘Nasal’), the nasal septum is apparently only partially mineralized, as is true for some other ankylosaurians (Witmer & Ridgely, 2008).

#### Pterygoid

The pterygoid comprises a main body and a rostral process. It is visible mainly in ventral aspect, but the caudal edge of the main body is visible in caudal aspect. The pterygoids do not contact one another caudally near the braincase; they are completely separated by the basisphenoid. In ventral aspect, the main body of the pterygoid forms a broad concave (very slightly convex dorsally) plate that is divided medially by the basisphenoid (Figs. 2C–2D and 3G–3H). The rostrolateral corner thickens where it contacts the ectopterygoid, but due to poor preservation, this contact is unclear. The lateral edges of the pterygoid arch ventrally, contacting the ventromedial portion of the pterygoid processes of the quadrate. The caudolateral corner of the pterygoid contacts the pterygoid ramus of the quadrate via a scarf joint. The caudal margin thins caudally. The quadrate process is dorsoventrally thin and caudolaterally directed. A small, circular foramen is present on the right pterygoid, close to the rostral edge and approximately a third of the length of the element from its medial edge. The corresponding area on the left pterygoid is obscured.

The rostral (vomerine) process of the pterygoid arises from the rostral edge of the main body of the pterygoid, most likely near the junction with the ectopterygoid (Figs. 2C and 2D). The processes are approximately 30% longer rostrocaudally than at its widest point transversely. The rostral processes arch medially to meet at the midline approximately level with the orbit, thus forming an interpterygoidal vacuity over the parasphenoid. The rostral processes of the pterygoids are closely associated with the parasphenoid, and appear to have closely neighboured it laterally. This is evident along the medial edge and surface, which parallel the fusiform shape of the parasphenoid. Due to poor preservation, it is unclear where the pterygoid-palatine contact is situated.

**Figure 6:**
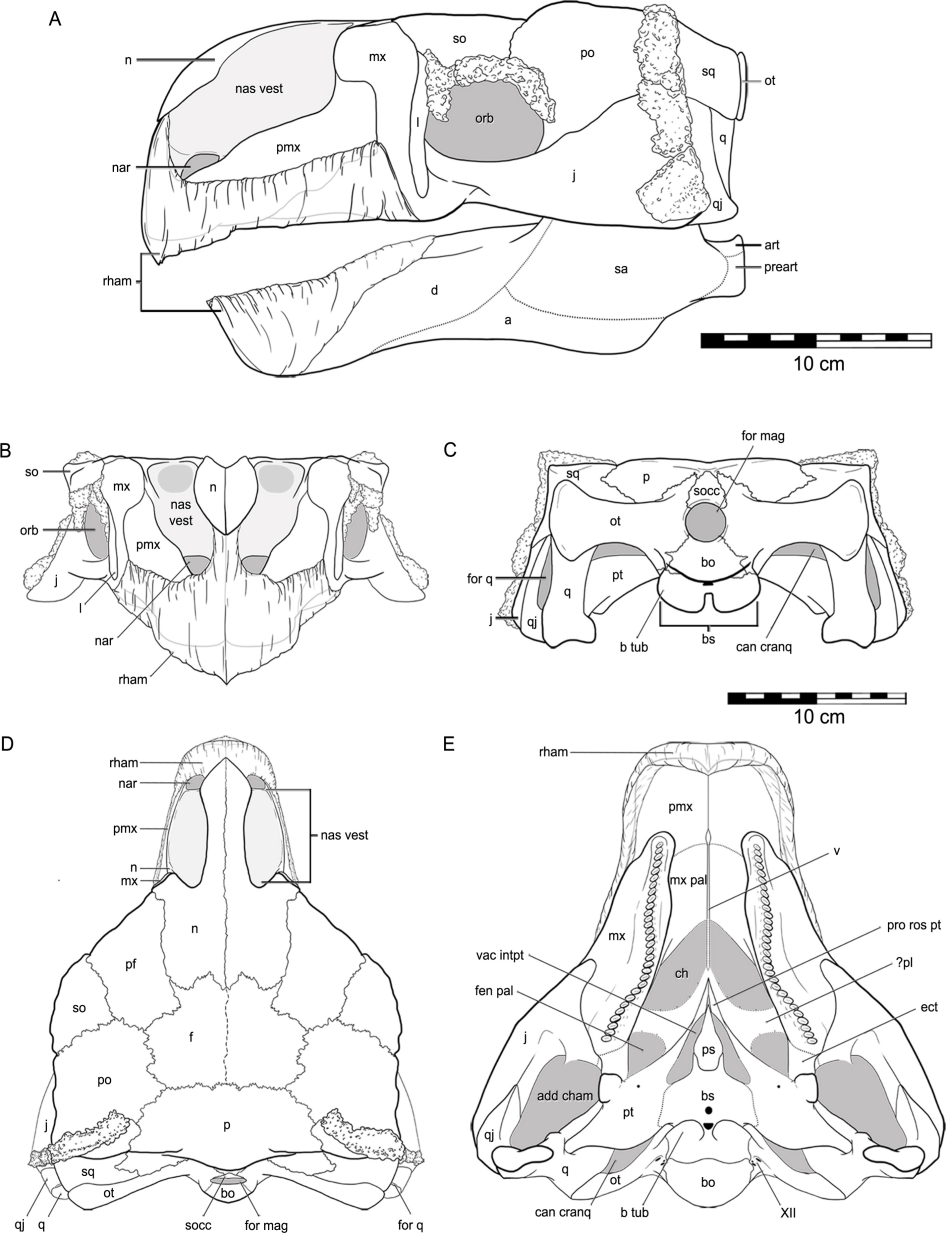
Reconstruction of the skull of *Kunbarrasaurus ieversi* gen. et sp. nov. (QM F18101), in (A) left lateral, (B) rostral, (C) caudal, (D) dorsal and (E) ventral aspects. Abbreviations: a, angular; art, articular; d, dentary; for q, quadrate foramen; nar, nares; pr art, prearticular; rham, rhamphotheca; sa, surangular. Legend: grey, void within skull; dashed line, partially fused suture; dotted line, inferred suture; grey line, bone outline or suture beneath rhamphotheca. Portions not known include rhamphotheca, apex of maxillary rostrum, caudal extent of maxillary secondary palate, ventral edges of jugal, quadratojugal and quadrates. Scale bars equal 10 cm.

The rostral (vomerine) processes of the pterygoid were originally thought to be the palatines, since in ankylosaurians the palatine and pteryoid form a broad, rostrally extensive shelf (‘caudoventral’ secondary palate) that obscure the parasphenoid from view. However compared with other dinosaurs, particularly stegosaurians and other ornithischians, the structures seen in QM F18101 are more similar to the rostral processes of the pterygoids and will thus be interpreted as such herein (Fig. 6E). Unfortunately CT resolution is poor in this area such that it cannot be utilised to verify this interpretation.

#### Ectopterygoid

The area in which the ectopterygoid is most likely to exist (i.e., between the maxilla, pterygoid and jugal) is highly fractured, thus the boundaries for this element are unclear, but are visible in some parts of the CT data (Fig. 5F). It may be fused to the maxilla. We provisionally assign this area as the ectopterygoid, and suggest possible sutural margins for the element (Fig. 6E)

#### Quadrate

The quadrates are nearly vertically orientated, rostrocaudally flattened and are very slightly concave rostrally and convex caudally (Figs. 2C–2D and 3G–3H). The articular ends flare laterally from the shaft. CT imagery reveals that the mandibular-articular (ventral) end is wider than the squamosal-articular (dorsal) end, and that the latter is more rounded. The quadrate is not coossified to the squamosal or the pterygoid (Figs. 3G–3H and 5E). The quadrate body shows a smooth-surfaced notch located in the lateral surface of the distal half of the quadrate and, with the adjacent quadratojugal, would form a quadrate foramen (see quadratojugal). This is confirmed with the CT imagery. In ventral aspect, the main body of the quadrate contacts the quadratojugal laterally, and forms the caudal margin of the subtemporal fossa. The pterygoid process of the quadrate extends rostrally and contacts the pterygoid medially and rostrally. The pterygoid process of the quadrate is thin and vertically orientated and forms the majority of the medial margin of the subtemporal fossa.

### Braincase region

#### Supraoccipital

The supraoccipital bone was adequately described in Molnar (1996a). The supraoccipital is situated on the caudal surface of the skull, directly dorsal to the foramen magnum, overlying the parietal and the otoccipitals (Figs. 3G and 3H). The supraoccipital crest is more prominent and thicker than in most ankylosaurians (Vickaryous, Maryańska & Weishampel, 2004; Miyashita et al., 2011).

#### Otoccipital

As in virtually all posthatchling archosaurians, including other ankylosaurians, the exoccipital and opisthotic elements are indistinguishably fused into a single compound element referred to as the otoccipital (Sampson & Witmer, 2007). The otoccipital dominates the occiput (Figs. 3G and 3H). The paroccipital processes extend to almost the entire caudal width of the skull. In ankylosaurians they are not usually as laterally extensive, although much of the caudal breadth of the occiput in more advanced ankylosaurians is conferred by the squamosal and quadratojugal horns and the nuchal and postocular caputegula (Blows, 2001; Arbour & Currie, 2013). The paroccipital processes are also separate from the adjacent bones, whereas in other ankylosaurians they are often fused to the squamosal and quadrate (Vickaryous, Maryańska & Weishampel, 2004). Medially, the otoccipital diverges around the foramen magnum, forming its dorsolateral, lateral and ventrolateral borders and contributing to approximately half of its circumference. The dorsal branch of this part of the otoccipital contacts the parietal both dorsally and laterally. The otoccipital overlaps the medial-most part of the caudal surface of the parietal. The medial-most part of the otoccipital in turn is overlain dorsally by the supraoccipital. The ventral part of the otoccipital diverges into rostral and caudal arms, separated from each other by a teardrop-shaped depression. This depression is situated immediately dorsolateral to the basioccipital–basisphenoid contact. The caudal arm is the only part of the otoccipital that contacts the basioccipital, from the foramen magnum to the caudal edge of the aforementioned depression. The rostral arm attaches to the basisphenoid, dorsolaterally to the basal tubera at approximately half its rostrocaudal length, and spans to the rostral edge of the depression. The teardrop-shaped depression houses three foramina for the hypoglossal nerve on its left lateral side but only one foramen is apparent on the right side. Unfortunately the latter part of the bone is damaged. The internal portion of the otoccipital is only visible in the CT scan data. The suture with the prootic is discernible in some areas (Fig. 5E), and together the two bones contain the endosseous labyrinth of the inner ear, which is relatively enormous and different from other ankylosaurians (indeed other archosaurians; see below).

#### Prootic

The prootic is visible only in the CT scan data (Figs. 5D–5F). The major contacts of the prootic are similar to those of other archosaurians (Romer, 1956), and sutures are visible with the otoccipital caudally, laterosphenoid rostrally, and parietal dorsally. Its suture ventrally with the basisphenoid cannot be made out in the CT data, but it is likely obliterated, as we have found in our studies of other posthatchling archosaurians (e.g., Sampson & Witmer, 2007; Witmer et al., 2008). As noted above, the prootic houses the rostral portion of the endosseous labyrinth of the inner ear. The canal for the facial nerve (CN VII) is discernible on both sides passing through the prootic just rostral to the labyrinth (Figs. 10A–10C), and the vestibulocochlear nerve (CN VIII) is present just caudal to the facial nerve, at least on the right side passing from the endocranial surface to the labyrinth of the inner ear. As is typically the case in archosaurians (Romer, 1956), the prootic forms the caudal border of the aperture for the trigeminal nerve (CN V) (Figs. 10A–10C), with the laterosphenoid completing it rostrally. The trigeminal foramen is unusual in being very long dorsoventrally (see below) (Figs. 10A–10C). Farther dorsally, the prootic borders the dorsal head vein foramen which is also bounded by the laterosphenoid and parietal. The dorsal head vein foramen has been reported in a varied assortment of dinosaurs (theropods: Sampson & Witmer, 2007; sauropods: Witmer et al., 2008; hadrosaurs: Evans, Ridgely & Witmer, 2009), but this is apparently its first record in ankylosaurians.

#### Laterosphenoid

The laterosphenoid is visible only in the CT scan data (Fig. 5E). As noted, the laterosphenoid contacts the prootic, parietal, and frontal with a clearly visible suture, particularly where it borders the trigeminal and dorsal head vein apertures. It is relatively thick dorsally but becomes a relatively mediolaterally thin and rostrocaudally narrow splint as it passes ventrally to reach the basisphenoid. As in other archosaurians that ossify an orbitosphenoid element (e.g., many theropods, sauropods; Sampson & Witmer, 2007; Witmer et al., 2008) the laterosphenoid forms the caudal margin of the orbitocerebral foramen and the apertures for the eye muscle nerves, which are not completely resolved in QM F18101.

#### Orbitosphenoid

The orbitosphenoid is obscured by matrix and only visible in the CT scan data (Fig. 5E). Some (e.g., Tumanova, 1987) have suggested that ankylosaurians may have multiple ossifications, such as sphenethmoid, orbitosphenoid, and presphenoid, but there is little evidence that these elements are distinct (Vickaryous, Maryańska & Weishampel, 2004). Indeed, all these elements are variable mineralisations or ossifications of the orbital cartilages and interorbital septum in archosaurians where they often bear different names (Sampson & Witmer, 2007). No distinctions can be discerned in QM F18101, and we regard the ossification as the orbitosphenoid. In this specimen, the element is a relatively thin element caudally but becomes thicker rostrally where it floors the olfactory tract. The sutures of the orbitosphenoid with adjacent elements (laterosphenoid, frontal, basisphenoid) are indistinct in most areas, although the suture with the basisphenoid is discernible in some areas. Part of the obscurity of the suture relates to the fact that the orbitosphenoid was apparently not as fully ossified or mineralized as other elements, which is also fairly common in archosaurians (Sampson & Witmer, 2007). As noted above, the orbitosphenoid shares with the laterosphenoid the apertures for the orbitocerebral vein and eye muscle nerves. The conjoined aperture for the paired optic nerves (CN II) is located at the base of the orbitosphenoid, and the basisphenoid forms the ventral border of the aperture.

#### Basioccipital

The external ventral surface of the basioccipital is discussed in detail by Molnar (1996a). However, contrary to Molnar (1996a), the basioccipital is not fused to the basisphenoid; the suture between the two elements is situated caudal to the basal tubera (Figs. 2C and 2D). Two very small nutrient foramina are present approximately halfway on the right side ventral surface of the basioccipital, with one foramen being directly ventral to the other. A crust of calcite mineralisation obscures the area where these foramina occur on the left side of the basioccipital. The occipital condyle lacks a neck.

#### Basisphenoid

As just noted, contrary to Molnar (1996a), the suture between the basisphenoid and basioccipital is visible—caudal to the basal tubera (Figs. 2C and 2D). The otoccipital-basisphenoid contact is also visible (see above) (Figs. 3G and 3H). In ventral aspect (Figs. 2C and 2D), at the rostral extremity of the basisphenoid are two peg-like rostrally-orientated projections, which laterally encase the parasphenoid. The flat surface of the basisphenoid rostral to the basal tubera is roughly pentagonal in outline and orientated slightly rostrodorsally. The basipterygoid processes of the basisphenoid are just rostral to the basal tubera and are laterally directed. Due to fracturing, it is unclear whether these processes are fused to the medial parts of the pterygoids. A small round nutrient foramen occurs on the median plane, immediately rostral to the basal tubera. There is a depression between the basal tubera, and within this hollow is a semicircular nutrient foramen. The basal tubera fan laterally to freely arch over the area in which the otoccipital contacts the basisphenoid. Caudal to the basal tubera the basisphenoid curves steeply dorsally, towards the occipital condyle. A small round nutrient foramen occurs on this caudal facing surface. The CT scan data reveals the structure of the pituitary fossa within the basisphenoid. The pituitary fossa is moderately large and is directed caudoventrally from the infundibular region where it joins the endocranial cavity. The cerebral carotid artery canals traverse the basisphenoid to enter the pituitary fossa about one-third of the distance from its caudoventral terminus. These structures are discussed further below.

#### Parasphenoid

The parasphenoid is a fused element, which projects rostrally from the basisphenoid along the midline between the interpterygoid vacuity (Figs. 2C and 2D). It has a bulky, fusiform shape and its circumference increases caudally, resulting in a bulging towards the caudal extremity. This bulge decreases slightly at the caudal extremity to accommodate the contact with the basisphenoid.

### Mandible

The preserved portions of the left mandibular ramus (dentary + coronoid process) are described in detail by Molnar (1996a) and will not be commented on further here. Figure 6A presents a restoration of the mandible in lateral aspect.

### Dermal ossifications

The majority of the dermal ossifications of QM F18101 were described in Molnar (1996a) and Molnar (2001b). Of note, the majority of the dermal ossifications on the skull are generally flat. QM F18101 does not possess any spines, horns or ‘boss-like’ dermal ossifications on its skull. The dermal ossifications with the most topography are those of squamosal-postorbital region, which have shallow keels. The largest of the keels occurs on the most ventral of these. This dermal ossification is missing a ventral portion, however the end of the keel is complete suggesting that it is only missing the ventral edge. Thus it appears that QM F18101 does not possess a quadratojugal or squamosal horn or ‘boss’.

The dermal ossifications of the orbit and nasal regions were not discussed by Molnar (1996a), thus they are described herein. Three dermal ossifications are associated with the dorsal half of the orbit (Figs. 3A–3D). These are most clearly seen in left lateral aspect, as all three are not fused to the supraorbital and postorbital bones. The most rostral dermal ossification (D1) is a thin, quadrilateral-shaped element with its long axis aligned at approximately 45° to the rostrolateral edge of the supraorbital. The middle dermal ossification (D2) is very slightly crescentic in shape. It lies directly ventral to D1, dorsoventrally along the caudal edge of the lacrimal. The most caudal dermal ossification (D3) has a crescentic outline and lies along the ventral edge of the supraorbital and the rostral edge of the postorbital. A similar series of ossifications on the right lateral side of the skull differs slightly from those on the left. D1 and D3 on the right are fused to supraorbital and the postorbital, respectively, whereas D2 remains detached. These elements may not be analogous to the ‘anterior and posterior supraorbital caputegulum’ (Arbour & Currie, 2013) as they occur within the orbit, ventral to the supraorbital and rostroventral to the postorbital. The anterior and posterior supraorbital caputegulum of all other ankylosaurians occur on the lateral edge of the skull roof, dorsolateral to the supraorbital.

The oblong dermal ossification that occurs in the right nasal vestibule (Figs. 3C and 3D) may resemble the condition seen in *Pinacosaurus* (Maryańska, 1977), particularly the dermal ossification that occurs more laterally to the ‘lateral osteodermal mass’ that caps the premaxillary-nasal region in *Pinacosaurus grangeri* (IVPP V16853) (Burns et al., 2011: Fig. 2). The exact *in vivo* position of this element, however, is unclear.

**Figure 7:**
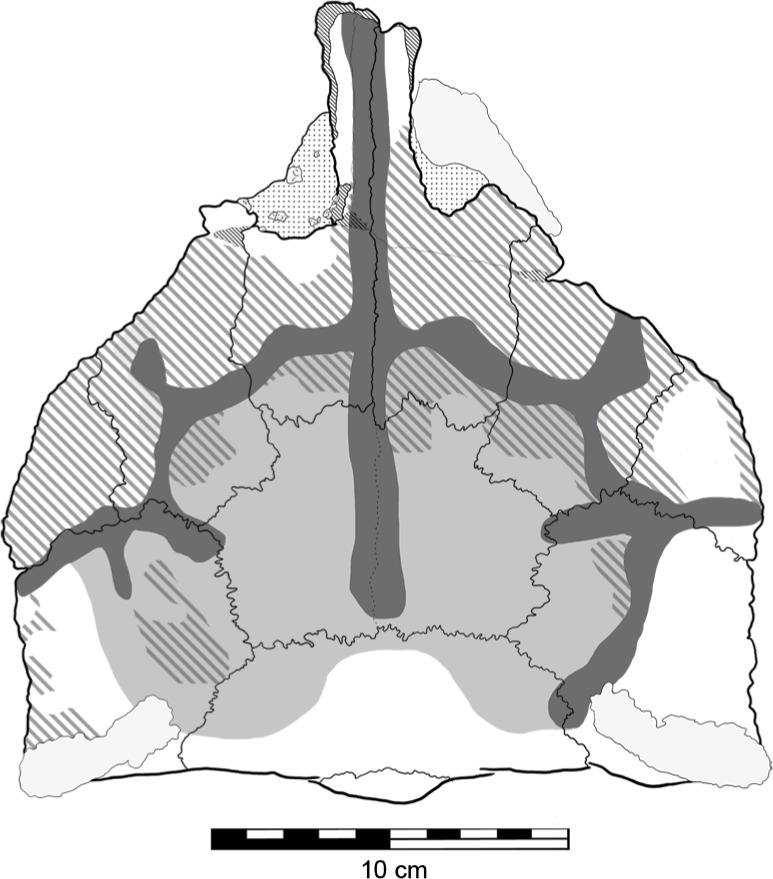
Sulci and evidence of periosteal osteogenesis on the dorsal surface of *Kunbarrasaurus ieversi* gen. et sp. nov. (QM F18101). Sulci network (dark grey); central depression (light grey); periosteal osteogenesis (grey stripe). Scale bar equals 10 cm.

Previously described by Molnar (1996a) are irregular ‘grooves’ or sulci on the dorsal surface of the skull (Fig. 7), which he presumed to be impressions caused by epidermal plates/scutes, most likely the ‘caputegulae’ described in other ankylosaurians (Blows, 2001; Arbour & Currie, 2013). If QM F18101 did possess these dermal ossifications, their lack of preservation may be explained by them not being ossified (i.e., keratinous) or not being co-ossified to the skull (Vickaryous, Russell & Currie, 2001; Arbour et al., 2013). These sulci are generally deeper on the right side of the skull than they are on left (Fig. 7). Additionally, the central part of the skull table, the frontals and the area immediately surrounding it, are slightly depressed but not to the extent of the sulci. Surrounding these sulci, parts of the supraorbitals, prefrontals, nasals and postorbitals have a rugose texture. Patches of this rugose texture are also found within the depressed area but not within the sulci (Fig. 7). This most likely represents reworking of the bone (‘periosteal osteogenesis’ Carpenter et al., 2001).

### Nasal cavity

**Figure 8:**
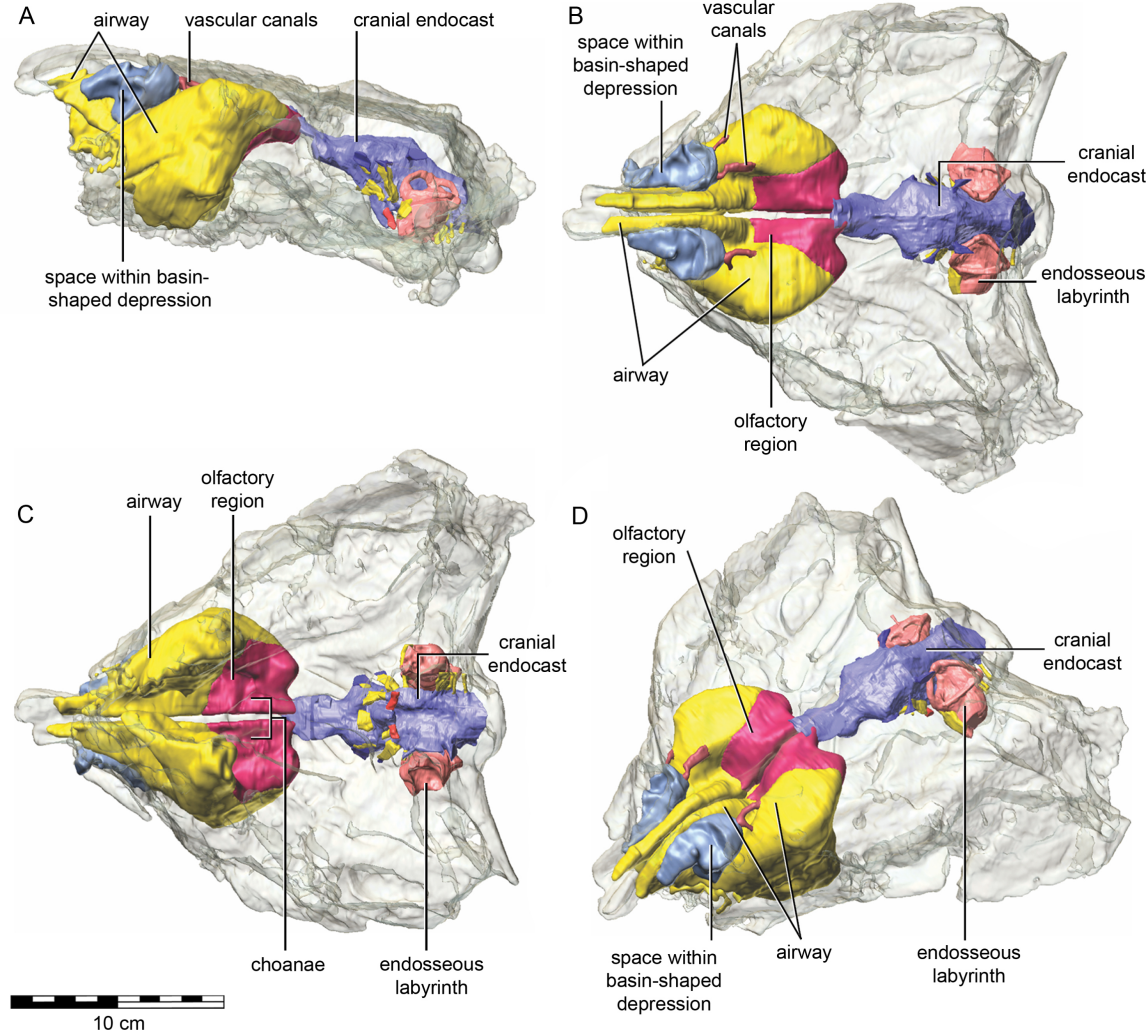
Semitransparent skull of *Kunbarrasaurus ieversi* gen. et sp. nov. (QM F18101) showing the position of components of the nasal cavity, cranial endocast, and endosseous labyrinth of the inner ear in (A) left lateral aspect, (B) dorsal aspect, (C) ventral aspect, and (D) left rostrodorsolateral aspect. Scale bar equals 10 cm.

The nasal cavity of QM F18101 presents some challenges to interpret given that (1) ankylosaurians in general have unusual and highly divergent nasal cavities that loop through the snout (Witmer & Ridgely, 2008: 1379 & 1383; Miyashita et al., 2011), (2) QM F18101 likely has a fairly basal phylogenetic position within Ankylosauria such that it is not clear to what extent the nasal morphology of other ankylosaurians applies to QM F18101, and (3) QM F18101 is incomplete rostrally and the CT scan data are often equivocal. These caveats aside, we here attempt to describe the major features of the nasal cavity based on digital segmentation of the CT scan data (Fig. 8, see also Supplemental Information 1).

**Figure 9:**
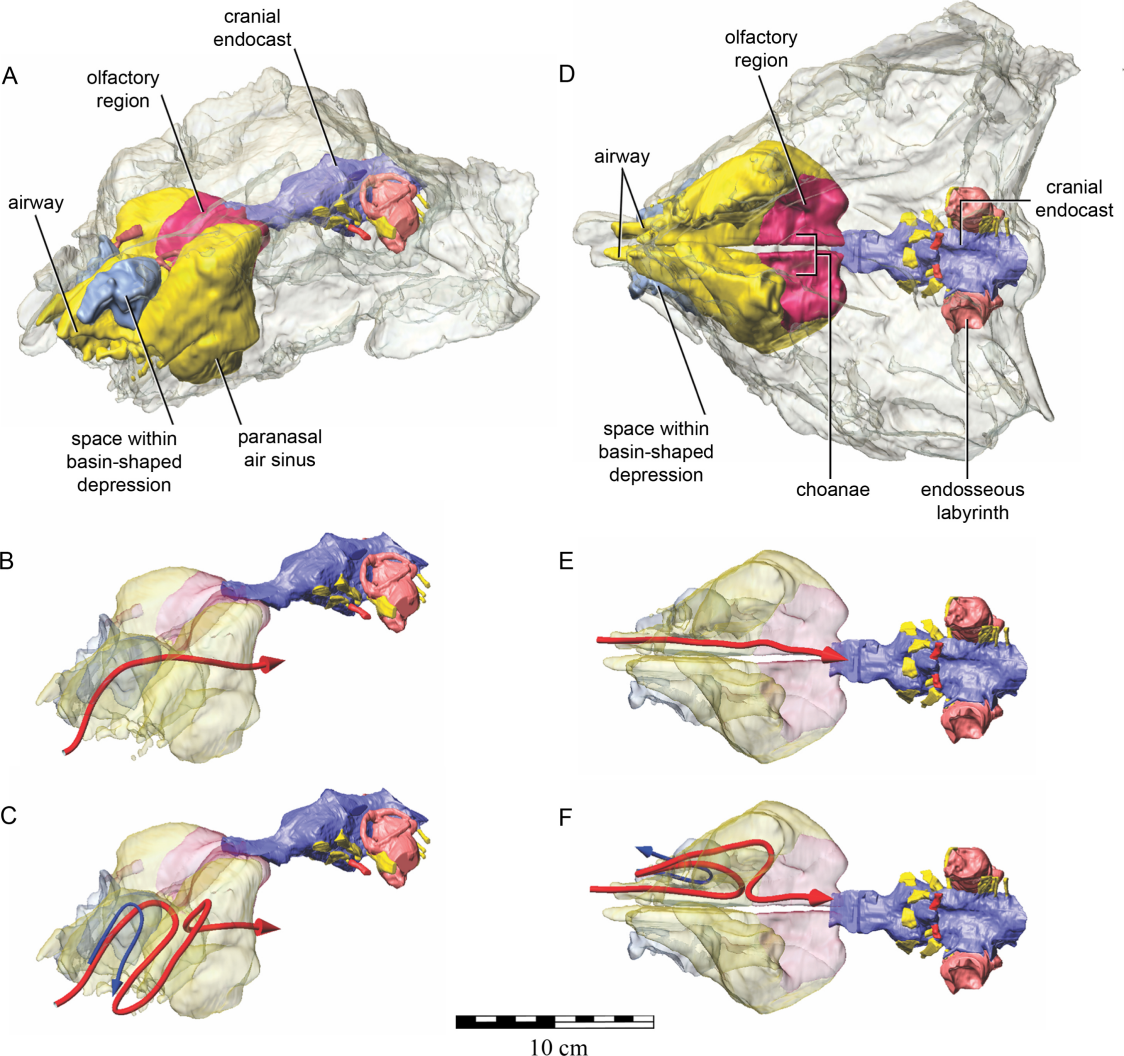
Semitransparent skull of *Kunbarrasaurus ieversi* gen. et sp. nov. (QM F18101) showing two hypotheses for the course of the nasal airway. Semitransparent skull of *Kunbarrasaurus ieversi* gen. et sp. nov. (QM F18101) showing the position of components of the nasal cavity, cranial endocast, and endosseous labyrinth of the inner ear in (A) left rostrodorsolateral aspect, and (D) ventral aspect. Two hypotheses for the course of the nasal airway are shown: (B) and (E) show the primitive, non-looping airway hypothesis in left rostrodorsolateral and ventral aspects, respectively; (C) and (F) show the derived looping airway hypothesis in left rostrodorsolateral and ventral aspects, respectively. The red arrow shows the hypothesized course of the main airway from nostril to choana, whereas the blue arrow shows the course of the putative loop passing the through the space within the basin-shaped depression of the nasal bone. Scale bar equals 10 cm.

As in other vertebrates, three main parts of the nasal cavity can be identified: the vestibule, the nasal cavity proper, and the olfactory region. The rostral portion of the skull is missing, and thus the full extent of the nasal vestibule cannot be restored. The vestibule is largely bounded by the premaxilla and nasal bones, both of which are better preserved on the left side. Not enough of the premaxillae are preserved to determine whether the pattern of premaxillary air sinuses and narial apertures described for *Pinacosaurus* (Hill, Witmer & Norell, 2003) were present. Likewise, much of the looping of the airway that was described as passing partly through the nasal vestibule in *Panoplosaurus* and especially *Euoplocephalus* (Witmer & Ridgely, 2008) cannot be fully assessed in QM F18101 due to incomplete preservation. Nevertheless, enough is preserved to suggest that QM F18101 had a more complicated airway than did typical non-ankylosaurian dinosaurs (Witmer, 1997; Witmer, 2001; Sampson & Witmer, 2007; Witmer & Ridgely, 2008; Bourke et al., 2014) (Fig. 9). The position of the nostril cannot be known precisely due to absence of the rostral-most portion of the snout, but it was presumably located rostroventrally as in other ankylosaurians (Hill, Witmer & Norell, 2003; Witmer & Ridgely, 2008), and indeed most other dinosaurs and amniotes in general (Witmer, 2001). The choanae opens into the oral cavity caudal to the maxillary secondary palate (Fig. 8C).

**Figure 10:**
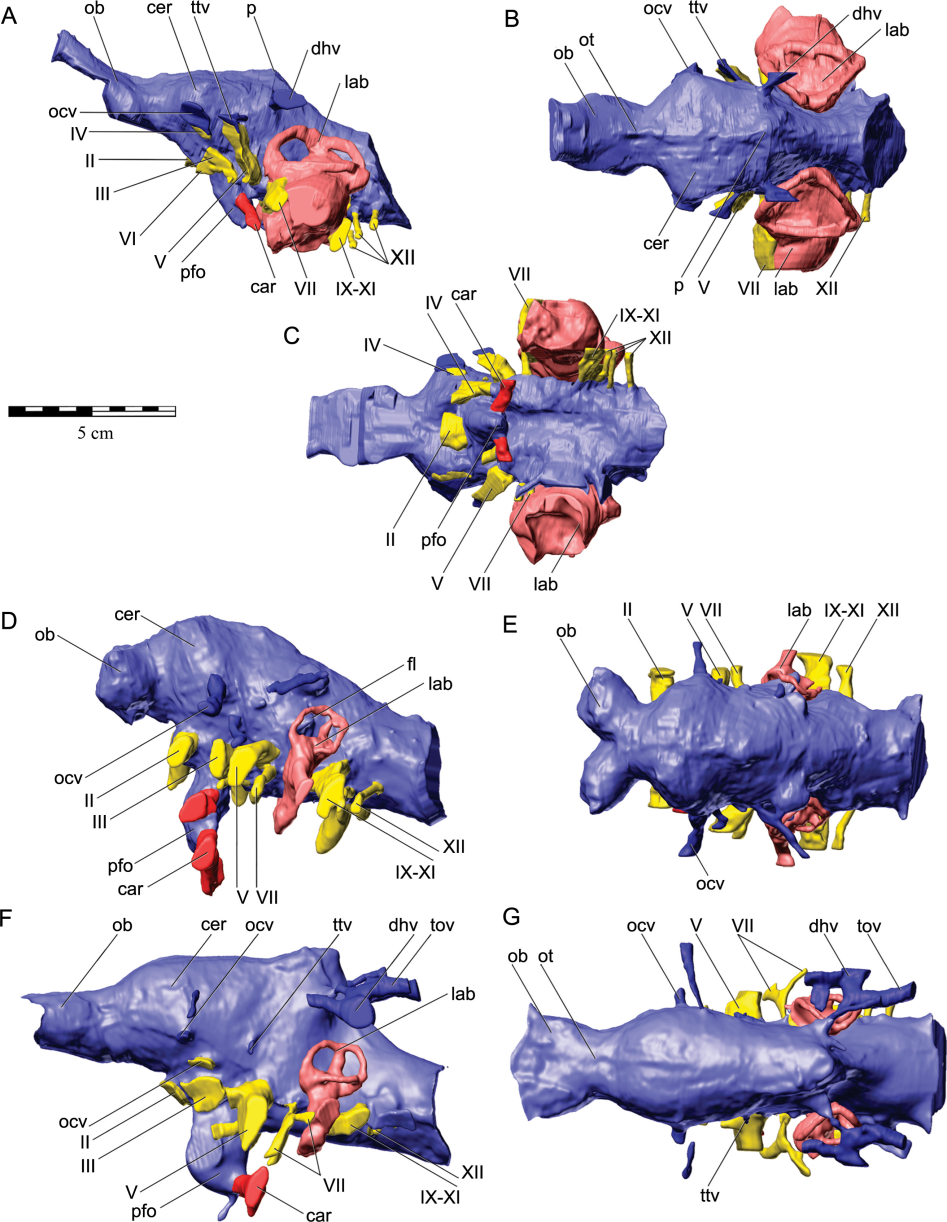
Cranial endocasts with endosseous labyrinths of the inner ear of (A–C) *Kunbarrasaurus ieversi* gen. et sp. nov. (QM F18101); (D), (E) *Euoplocephalus tutus* (AMNH 5405); and (F, G) *Stegosaurus stenops* (CM 106); in left lateral aspect (A, D, F) dorsal aspect (B, E, G) and ventral aspect (C). Abbreviations: car, cerebral carotid artery; cer, cerebral hemisphere; dhv, dorsal head vein; fl, flocculus of cerebellum; lab, endosseous labyrinth; ob, olfactory bulb; ocv, orbitocerebral vein; ot, olfactory tract; p, pineal; pfo, pituitary fossa; tov, transverso-occipital (caudal middle cerebral) vein; ttv, transversotrigeminal (rostral middle cerebral) vein; II, optic nerve; III, oculomotor nerve; IV, trochlear nerve ; V, trigeminal nerve; VI, abducens, nerve; VII, facial nerve; VIII, vestibulocochlear nerve ; IX–XI, vagal (metotic, jugular) canal transmitting the glossopharyngeal, vagus, and accessory nerves; XII, hypoglossal nerve. Scale bar equals 5 cm.

The course of the airway between the nostril and choanae is not entirely clear but some options are presented here (Fig. 9). As shown in Figs. 8 and 9, the main airway passed from the nostril region to run dorsomedially adjacent to the nasal septum (which is discernible in QM F18101, even if incompletely mineralized and preserved, as noted above). The airway passed dorsally over the maxillary secondary palate to reach a large open chamber, comprising the nasal cavity proper, bounded by the nasal and frontal bones dorsally, the maxilla, premaxilla, and lacrimal laterally, and maxilla ventrally. The medial portion of the chamber is presumably the olfactory recess (coloured red in Figs. 8 and 9), given its location adjacent to the olfactory bulb of the brain in the typical cul-de-sac caudodorsomedial to the choana (Witmer & Ridgely, 2008). There are no olfactory turbinates preserved in QM F18101 to confirm this functional segregation of the nasal cavity proper, but this relationship is highly conserved evolutionarily, and other ankylosaurians have been described with olfactory turbinates in this region (Maryańska, 1977; Witmer & Ridgely, 2008). The main respiratory (i.e., non-olfactory) airway passed more laterally into the nasal cavity proper (Fig. 9).

From this chamber, the airway may simply have passed ventromedially to reach the choana. In this non-looping-airway hypothesis (Figs. 9B and 9E), the open space extending rostrally above the maxillary secondary palate could be regarded as part of the nasal cavity proper and the space within the maxilla above the tooth row would be a paranasal sinus homologous with that of the antorbital sinus of other archosaurians, which would be consistent with earlier interpretations of ankylosaurians nasal cavities (e.g., Maryańska, 1977; Witmer, 1997; Vickaryous, Maryańska & Weishampel, 2004), prior to the discovery of the looping airways of both nodosaurids and ankylosaurids (Witmer & Ridgely, 2008). Again, given the potentially very basal position of QM F18101, a more plesiomorphic condition of the nasal cavity cannot be ruled out on phylogenetic grounds. However, a looping-airway hypothesis is also tenable (Figs. 9C and 9F), in which case the rostral space above the secondary palate could have transmitted a loop of the airway. Supporting the looping-airway hypothesis is the rostrally-facing, basin-shaped structure in the nasal bone described above (Figs. 5B–5C and 8). The nasal basin is hard to interpret as anything other than as a structure housing some portion of the nasal cavity. The space within the basin is walled off from the dorsomedial tract of the main airway by the nasal bone (Figs. 5B and 5C), suggesting that the nasal basin housed either a novel paranasal air sinus within the nasal bone or a loop of the airway within the nasal vestibule (Figs. 9C and 9F). Unfortunately, the front portion of the snout that would have housed these nasal loops or sinuses is not preserved, and thus we cannot know how the space within the nasal basin connected with other components of the nasal air spaces. Likewise, in the looping-airway hypothesis, it is not clear how the airway would loop back to the choana, and it is necessary to invoke that the airway looped both fore and aft through the space above the secondary palate (Figs. 8C and 8F), which is reasonable but for which there is no osteological evidence at this time. Thus, although the precise course of the airway in QM F18101 is not clear at present, there is at least some evidence (e.g., the nasal basin, partial partitioning of the nasal cavity proper) that some of the complexity of the nasal cavity that characterized more derived ankylosaurians (Witmer & Ridgely, 2008) had evolved already in QM F18101.

### Cranial (brain) endocast

A cranial endocast of QM F18101 was generated from the CT scan data (Figs. 10A–10C, see also Supplemental Information 1) and provides information on brain structure in QM F18101. As is typically the case for reptiles (Hopson, 1979; Witmer et al., 2008), the neural tissue of the brain apparently did not fill the endocranial cavity and thus the endocast is a reflection of the dural envelope more so than the brain itself. The cranial endocast has been presented previously for a range of ankylosaurians, including *Euoplocephalus* (Figs. 10D and 10E, herein) (Coombs, 1978b; Hopson, 1979; Witmer & Ridgely, 2008; Miyashita et al., 2011), *Polacanthus* (Norman & Faiers, 1996), *Struthiosaurus* (Pereda Suberbiola & Galton, 1994), *Panoplosaurus* (Witmer & Ridgely, 2008), and *Hungarosaurus* (Ősi, Pereda Suberbiola & Földes, 2014). The endocast of QM F18101 is generally well preserved with little to no evidence of distortion. It is similar to those of other ankylosaurians but has some important general differences. For example, the length of endocast represents approximately 40% of QM F18101’s total skull length, whereas the endocast of *Euoplocephalus* represents approximately 25% of skull length. Also, the endocast of QM F18101 has some unusual attributes that probably represent incomplete ossification of the bones walling in the endocranial cavity, whereas those of other ankylosaurians are extremely well ossified (Vickaryous, Maryańska & Weishampel, 2004; Witmer & Ridgely, 2008; Miyashita et al., 2011; Ősi, Pereda Suberbiola & Földes, 2014).

The paired olfactory bulbs in QM F18101 are modestly developed and apparently contact each other at the midline. This latter point represents the primitive condition (as characterized by *Stegosaurus*; Figs. 10F and 10G; see also Galton, 2001) and contrasts with that of other ankylosaurians in which the olfactory bulbs are strongly divergent from the midline, as in *Euoplocephalus* (Figs. 10D and 10E; see also Hopson, 1979; Miyashita et al., 2011), *Panoplosaurus* (Witmer & Ridgely, 2008), and, to a lesser extent *Hungarosaurus* (Ősi, Pereda Suberbiola & Földes, 2014). As in other ankylosaurians just noted (see also Pereda Suberbiola & Galton (1994) on *Struthiosaurus*), the olfactory tract is relatively short. The cerebral hemispheres are clearly indicated on the endocast as lateral swellings in the rostral portion of the endocast, but distinct optic lobes and cerebellum are not grossly observable, which is typical of other ankylosaurians (Hopson, 1979; Witmer & Ridgely, 2008; Miyashita et al., 2011; Ősi, Pereda Suberbiola & Földes, 2014). The endocast of QM F18101 does not have a floccular lobe of the cerebellum, which is the condition in most ankylosaurians, although two specimens of *Euoplocephalus* display a flocculus (Fig. 10D) (Hopson, 1979; Miyashita et al., 2011). The endocast shows a median dural peak that is in the approximate position of the pineal gland (epiphysis cerebri). The pineal has been identified in endocasts of *Euoplocephalus* (Coombs, 1978b; Miyashita et al., 2011), and so its presence in QM F18101 is perhaps not unexpected, but it is worth pointing out that a diversity of dinosaurs have a variety of median dural peaks, not all of which are likely to be epiphyseal in origin (Witmer et al., 2008; Witmer & Ridgely, 2009). As noted above, the pituitary fossa within the basisphenoid preserves the general disposition of the gland. Unlike any other ankylosaurian endocasts, in which the pituitary projects more or less directly vertically below the cerebral region, in QM F18101 the pituitary angles strongly caudally from the infundibular region such that it underlies the brainstem. The outgroup condition (e.g., *Stegosaurus*; Fig. 10F) is to have a more vertical pituitary, and thus QM F18101 would have a derived condition. Another derived condition of the pituitary is that the cerebral carotid arteries enter the pituitary fossa not at its distal terminus, as in *Stegosaurus* and other ankylosaurians, but rather enter at about one-third the length of the pituitary from the distal terminus.

Many of the cranial nerves and vascular structures can be identified in the CT data and are included in the digital endocast (Fig. 10). The optic nerves (CN II) apparently emerge together near the midline rather than being widely separated from each and directed more or less laterally, as in other ankylosaurians (Hopson, 1979; Miyashita et al., 2011; unpublished endocast of *Edmontonia*, AMNH 5831, done at Ohio University). QM F18101 displays the primitive condition in this regard, resembling *Stegosaurus* and most other dinosaurs (Hopson, 1979). In more advanced ankylosaurians such as *Euoplocephalus*, *Edmontonia*, and *Panoplosaurus* (L Witmer, pers. obs., 2015), the orbits and eyeballs are more caudally positioned within the skull, resulting in the lateral course of not just the optic nerves, but also the nerves supplying the extraocular muscles (CN III, IV, VI). The canals for these nerves in QM F18101 are not as discrete as in other ankylosaurians but what is preserved is consistent with QM F18101 retaining a more plesiomorphic condition, suggesting that, as with the optic nerves, the extraocular muscle nerves were not directed strongly laterally. As noted above, the side wall of the braincase is not as ossified as in other ankylosaurians (Vickaryous, Maryańska & Weishampel, 2004), and there are large gaps on either side of the laterosphenoid (Fig. 5E). Typically, the oculomotor (CN III) and trochlear (CN IV) nerves pass through foramina in the suture located between the laterosphenoid and orbitosphenoid, but in QM F18101 the corresponding region is a large open space without discrete canals. Cranial nerves III and IV presumably travelled through the ventral portion of the aperture, whereas the dorsal portion of the aperture probably transmitted the orbitocerebral vein, which is found in ankylosaurians, as well as other dinosaur clades (Witmer et al., 2008; Miyashita et al., 2011). The abducens nerve (CN VI) reaches the orbit by traversing a rough canal arising from the region of the brainstem, which is typical. It may merge with the laterosphenoid-orbitosphenoid aperture, but the CT scan data are not entirely clear on this point.

The trigeminal nerve (CN V) in archosaurians emerges through a foramen between the laterosphenoid and prootic (Romer, 1956) but in QM F18101, again, there is a large unossified gap rather than a discrete foramen (N.B. this is not an artefact of preservation). As a result, no distinctions can be made between the three branches of the trigeminal nerve. On the left side, a small canal traverses the braincase wall to pass from the endocranium to reach the laterosphenoid-prootic aperture; this canal almost certainly transmitted the transversotrigeminal vein (= rostral middle cerebral vein), in that in many archosaurians this vein opens into or just above the trigeminal foramen (Sampson & Witmer, 2007; Witmer et al., 2008; Witmer & Ridgely, 2009). The facial nerve (CN VII) is visible on both sides of QM F18101 passing through the prootic just rostral to the labyrinth of the inner ear. The vestibulocochlear nerve (CN VIII) is visible on the right side of the endocast, before disappearing into the inner ear. Better preserved on the left side, the vagal (= metotic, jugular) canal is present, and presumably transmitted the glossopharyngeal (CN IX), vagus (CN X), and accessory (CN XI) nerves, much as in other ankylosaurians and indeed other sauropsids (Romer, 1956; Sampson & Witmer, 2007). The left side of the endocast displays three canals, all of which we interpret to have transmitted branches of the hypoglossal nerve (CN XII). The number of hypoglossal foramina is variable in dinosaurs, but some specimens of *Euoplocephalus* and *Amtosaurus* have three (Averianov, 2002; Miyashita et al., 2011). Finally, the endocast of QM F18101 shows the course of the dorsal head vein, passing through an aperture between the prootic, laterosphenoid, and parietal to reach the adductor chamber in the temporal region. The dorsal head vein was present in *Euoplocephalus* (Figs. 10D and 10E; N.B. unlabelled in Fig. 7 of Miyashita et al., 2011) and, as noted above, a wide diversity of other dinosaurs.

### Endosseous labyrinth of the inner ear

**Figure 11:**
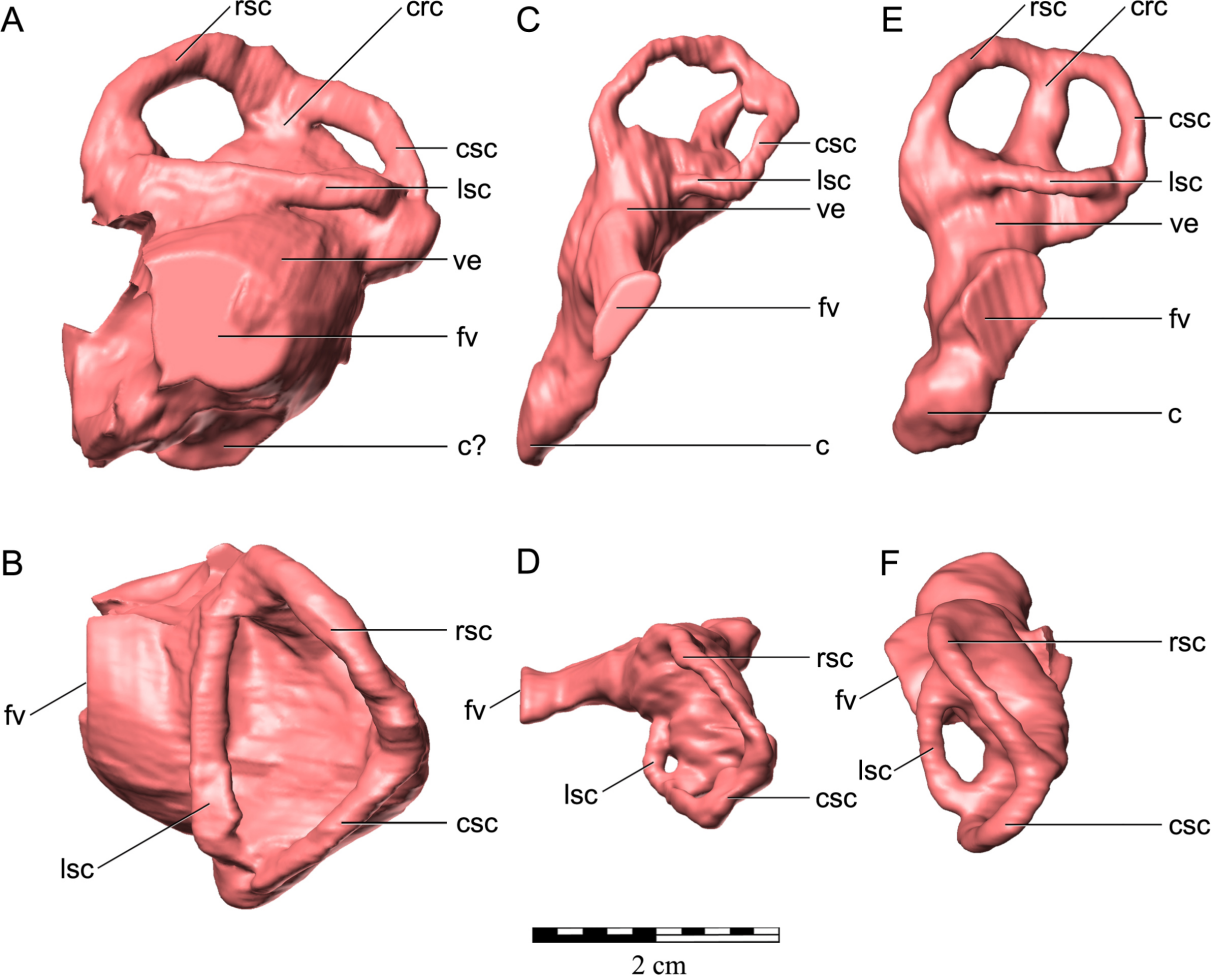
Endosseous labyrinths of left inner ears of (A, B) *Kunbarrasaurus ieversi* gen. et sp. nov. (QM F18101); (C, D) *Euoplocephalus tutus* (AMNH 5405); and (E, F) *Stegosaurus stenops* (CM 106); in left lateral aspect (A, C, E) and dorsal aspect (B, D, F). Note that although QM F18101 has a much shorter skull length, the endosseous labyrinth is about twice as large in its rostrocaudal and mediolateral dimensions than the other two species; its full dorsoventral dimension is not knowable because the ventral portion was not fully ossified. Abbreviations: c, cochlear canal; crc, crus communis; csc, caudal semicircular canal; fv, fenestra vestibuli (ovalis); lsc, lateral semicircular canal; rsc, rostral semicircular canal; ve, vestibular region. Scale bar equals 2 cm.

The endosseous labyrinth of the inner ear of QM F18101 (Figs. 10A–10C and 11A–11B) is proportionally enormous relative to both the size of the skull and the relative sizes of the labyrinths in other ankylosaurians (Witmer & Ridgely, 2008; Miyashita et al., 2011) (Figs. 10D–10E and 11C–11D). Despite the skull of QM F18101 (approx. 25 cm, rostrocaudally) being smaller than that of *Euoplocephalus* (AMNH 5337, 5405 and NHMUK R4947 approx. 40–50 cm, rostrocaudally) or *Panoplosaurus* (NMC 2759, ROM 1215 approx. 35 cm, rostrocaudally), the inner ear is much larger in absolute size (Fig. 11, see also Supplemental Information 1). Moreover, the inner ear is highly divergent in morphology, as well. For example, rather than being separated by bone from the endocranial cavity (as in basically all other archosaurians groups; L Witmer, pers. obs., 2015), in QM F18101 the prootic and otoccipital bones fail to enclose the inner ear medially such that the vestibular portion of the inner ear is broadly open to the endocranial cavity (Fig. 5E). Likewise, the ventral limit of the inner ear, which in other archosaurians would correspond to the cochlear (or lagenar) region, is also incomplete or poorly ossified, such that it, too, is largely open (Fig. 5F). These attributes are previously unreported in dinosaurs (if not all of Sauropsida). The morphology is bilaterally symmetrical and shows no overt signs of pathology. The functional consequences of these modifications are obscure. In dorsal aspect, the semicircular canals appear to form a triangle, with the rostral and caudal semicircular canals being angled at approximately 45° to the sagittal plane, rostrolaterally and caudolaterally, respectively, and together form slightly more than a 90° angle. The lateral semicircular canal runs parallel to the median plane and forms acute angles with each of the vertical canals (Fig. 11B). The crus communis is extremely short, probably due to the large size of the vestibular region, which also contributes to the semicircular canals all seeming very short. The vestibule is spherical, which is unlike any known dinosaur. This condition is more similar to that in turtles and *Sphenodon* (Walsh et al., 2009), although even in these clades the inner ear is more discretely enclosed by bone. The cochlear region of the inner ear is hard to interpret given that its ventral terminus is so poorly defined. The fenestra vestibuli (= ovalis) of QM F18101 is large, relatively round, and laterally orientated. No columella (stapes) is preserved, and, combined with the curious structure of the inner ear, the auditory apparatus thus remains obscure.

### Taxonomic status of QM F18101

Based on the clear number of osteological differences, and thus diagnostic characters, between the cranium of QM F18101 and other ankylosaurians (see ‘Diagnosis’ and discussed above) we consider the specimen known as *Minmi* sp. (QM F18101) sufficiently distinct from other ankylosaurian taxa to warrant referral to a new genus and species: *Kunbarrasaurus ieversi* gen. et sp. nov. Furthermore preliminary investigations into the postcranial elements of QM F18101 reveal that a number of significant differences exist between it and the holotype of *Minmi paravertebra* (QM F10329), but this is part of an ongoing study that will be presented elsewhere. Accompanying the description of the postcranium of *K. ieversi* will be an in-depth analysis into its phylogenetic position.

Of note, *Minmi paravertebra* (QM F10329) has recently being assigned to *nomen dubium* by Arbour & Currie (in press). As *Minmi paravertebra* comprises postcranial material only, an in-depth comparison and reappraisal of the specimen will be conducted in conjunction with the redescription of the postcranium of QM F1810; something that is beyond the scope of this study. Pending the forthcoming publication, we still consider *Minmi paravertebra* sufficiently different to other ankylosaurians, particularly within Australia and other Gondwanan landmasses, to represent a distinct genus. Consequently *Kunbarrasaurus *represents the second named genus of ankylosaurian from Australia.

## Discussion

The general shape of the skull of *K. ieversi* exhibits characteristics of both non-eurypodan thyreophorans and ankylosaurians (discussed above). Of those non-eurypodan thyreophoran and ankylosaurian taxa for which cranial sutures are visible, the overall arrangement of the cranial bones and general shape of the skull of QM F18101 is more similar to *Scelidosaurus harrisonii* (Owen, 1861) and *Cedarpelta bilbeyhallorum* (Carpenter et al., 2001) than it is to that of *Pinacosaurus* (Maryańska, 1977; Hill, Witmer & Norell, 2003). As previously discussed, phylogenetically QM F18101 (now *K. ieversi*) is typically recovered as a basal ankylosaurian (e.g., Thompson et al., 2012; Arbour & Currie, in press), which is consistent with its mosaic of derived and plesiomorphic morphological features. Based on the new assessment of the skull of *K. ieversi* presented here and the comparisons conducted, at this time the authors herein support the previously proposed positions of QM F18101 as a basal ankylosaurian (e.g., Hill, Witmer & Norell, 2003; Vickaryous, Maryańska & Weishampel, 2004; Leahey, Molnar & Salisbury, 2008; Leahey, Molnar & Salisbury, 2010; Thompson et al., 2012; Arbour & Currie, in press).

As one of the world’s most complete basal ankylosaurians, *K. ieversi* represents an ideal taxon with which to observe the evolutionary transition between non-eurypodan thyreophorans and ankylosaurians, such as fusion of sutures, closure of fenestrae, origin of cranial dermal ossifications and development of the unique nasal cavity and endocranial systems.

The pattern of fusion of the skull of *K. ieversi* appears to be similar to *Cedarpelta bilbeyhallorum* (Carpenter et al., 2001), with more of the caudally positioned elements being fused than those elements that are more rostrally positioned (Carpenter et al., 2001). However *K. ieversi* differs in that the frontals are partially fused and the bones of the basicranium and the postorbital and jugal are not fused. It is notable that in both taxa it is the sutures that have fused or are in the process of fusing, rather than becoming obscured by dermal ossifications. This pattern of fusion differs from that which is observed in some derived ankylosaurians (e.g., *Tarchia kielanae* Arbour, Currie & Badamgarav, 2014; *Pinacosaurus* Burns et al., 2011) where the more rostrally-positioned nasals and frontals are obscured by ornamentation. However the mode of fusion differs between *K. ieversi* and those of derived ankylosaurians (discussed below).

At least two scenarios have been proposed to explain the closure of the cranial fenestrae in ankylosaurians. Hill, Witmer & Norell (2003) and Parish (2005) have proposed that the closure is the result of expansions of surrounding cranial bones. Alternatively, Maryańska (1971) and Witmer & Ridgely (2008) have suggested that it is a consequence of an overall thickening of cranial bones and the covering of the fenestrae with osteoderms. The closure of the antorbital fenestrae in *K. ieversi* appears to be associated with an expansion of cranial bones, as there is no evidence of bone thickening and the skull possesses few dermal ossifications and none within the vicinity of the antorbital fenestrae. More specifically, the closure of the antorbital fenestrae in *K. ieversi* would have resulted from the caudal expansion of the maxilla and the ventral extension of the lacrimal, since the jugal is not as rostrally extensive as that of other thyreophorans (see Norman, Witmer & Weishampel, 2004; Vickaryous, Maryańska & Weishampel, 2004). Similarly, the closure of the supratemporal fenestrae in *K. ieversi* appears to have resulted chiefly from the expansions of the parietal and the postorbital, as the squamosal is restricted to the very caudal edge of the skull. The squamosal of other ankylosaurians is more extensive on the dorsal surface of the skull than that of *K. ieversi* (Maryańska, 1971; Maryańska, 1977; Godefroit et al., 1999; Carpenter et al., 2001; Hill, Witmer & Norell, 2003; Burns et al., 2011).

Current work on the dermal ossifications of the ankylosaurian skull suggests two types of ornamentation: epidermal ossification (osteoderms) and periosteal osteogenesis (Carpenter et al., 2001) (“dermatocranial elaboration” of Vickaryous, Russell & Currie, 2001). *K. ieversi* exhibits evidence of both of these forms of ossifications. Osteoderms are present in the narial region and around the dorsal rim of the orbit, specifically near the prefrontal–supraorbital suture dorsally and astride the postorbital–squamosal, squamosal–jugal and jugal–quadratojugal sutures. A sheet of ossicles also extends across from the dorsolateral areas of the nuchal region. Periosteal osteogenesis, represented by the areas of rugosity, is also present on the dorsal surface of the skull (described above—‘Dermal ossifications’). Unlike some ankylosaurians (e.g., *Cedarpelta* and *Pinacosaurus*; see Carpenter et al., 2001) the rugose texture on the skull of *K. ieversi* is generally not restricted to individual elements. Furthermore the cranial sutures are not affected by these rugosities, in fact the sutures are more apparent. It is unclear whether this may be an ontogenetic characteristic of the skull (as has been suggested for other taxa by Vickaryous, Russell & Currie, 2001). As other specimens come to light, *K. ieversi* may provide further evidence that the extensive dermal covering on the crania of more derived ankylosaurians arose under differing modes of ossification (Carpenter et al., 2001; Vickaryous, Russell & Currie, 2001; Arbour & Currie, 2013).

The basal position of *K. ieversi* in most phylogenetic analyses has made it difficult to interpret some of the potentially more divergent aspects of its morphology. For example, with regard to the nasal cavity, given that *K. ieversi* may represent an outgroup to ankylosaurian taxa known to have complicated looping nasal passages, it is phylogenetically ambiguous whether a looping airway should be expected. Unfortunately, the preserved morphology is no more definitive. A valid hypothesis is that *K. ieversi* lacked the nasal convolutions of more derived ankylosaurians and instead had a simpler airway that passed from nostril to choana, with a paranasal air sinuses that probably would be the homolog of the typically archosaurian antorbital sinus; this arrangement was in fact expected prior to the discoveries provided by CT scanning and 3D visualization (Witmer & Ridgely, 2008; Miyashita et al., 2011). However, another valid hypothesis is that *K. ieversi* indeed had a looping airway, with the primary evidence being the unique rostrally-facing, basin-shaped structure in the nasal bone. The latter structure is too incomplete to provide clear interpretations, but it certainly suggests novel anatomy in the nasal vestibule, and the airway is the chief candidate for producing that feature. Indeed, that basin-shaped structure could have housed a loop of the airway, although not enough is preserved to homologize it with airways of other ankylosaurians (Witmer et al., 2008). Thus, if the latter hypothesis is accepted, it may be argued that *K. ieversi* provides evidence that a convoluted nasal airway of some kind characterizes the entire ankylosaurian clade.

Although the nasal cavity may ultimately be shown to conform to known (albeit highly derived) morphology, some aspects of the braincase are so unusual that they defy comparison. For example, although the laterosphenoid—a synapomorphy of archosaurians (Clark et al., 1993) —is present in *K. ieversi*, the form of the laterosphenoid and orbitosphenoid is unlike that of other dinosaurs (Weishampel, Dodson & Osmólska, 2004) in that there are large unossified gaps between the bones rather than more extensive bony walls with discrete foramina. The prootic and otoccipital bones are also morphologically unique, which is reflected in the highly aberrant structure of the inner ear. Again, there are large unossified sections both medially and ventrally that are unlike any sauropsid, let alone dinosaur. In some ways, the braincase and inner ear is almost at an “embryonic” level of ossification in that even hatchling and very young extant sauropsids have completely ossified walls of the labyrinth. Obviously, we are not suggesting that QM F18101 is actually embryonic, but only use the comparison to highlight just how unusual this structure is. It is tempting to suggest that these unexpected attributes reflect paedomorphosis, and, indeed it cannot be ruled out.

## Conclusions

The assignment of a new genus and species name *K. ieversi* to QM F18101 (formerly known as *Minmi* sp.) is based on a significant number of features that distinguish it from other ankylosaurians. Many of the cranial sutures of *K. ieversi* have not fused, nor are they obscured by dermal ossifications. The closure of the antorbital and supratemporal fenestrae of *K. ieversi* is most likely due to the expansion of cranial bones, and not the result of overgrowth of dermal ossifications. The ornamentation of the skull of *K. ieversi* is the result of both epidermal ossification (osteoderms) and periosteal osteogenesis. Some aspects of the nasal cavity remain obscure, but there is enough evidence to suggest that *K. ieversi* had a more complicated airway than in non-ankylosaurian outgroups. It is presently unclear whether *K. ieversi* had a convoluted, looping nasal passage to the extent seen in more advanced ankylosaurians. Some aspects of the braincase are potentially unique among known species, such as the unusual inner ear, which is not only extremely large but also has a divergent morphology due to lack of ossification medially and ventrally.

## Supplemental Information

10.7717/peerj.1475/supp-1 Appendix S1 Appendices for Cranial osteology of Minmi sp

10.7717/peerj.1475/supp-2 Supplemental Information 1 3D pdf of endosseous cavities of Minmi sp

## Institutional Abbreviations

AM: Australian Museum
AMNH: American Museum of Natural History
CEUMP: Utah State University Eastern Prehistoric Museum (formerly Prehistoric Museum of College of Eastern Utah)
DMNH: Denver Museum of Nature and Science (formerly Denver Museum of Natural History)
MPC: the Mongolian Paleontological Center (formerly Geological Institute Section of Palaeontology and Stratigraphy)
HBV: Hebei College of Geology
IVPP: Institute for Vertebrate Palaeontology and Paleoanthropology
KUVP: Kansas University Vertebrate Paleontology
NHMUK: Natural History Museum
CMN: Canadian Museum of Nature (formerly National Museums of Canada)
PIN: Paleontological Institute, Russian Academy of Sciences
QM: Queensland Museum
ROM: Royal Ontario Museum
SMU: Southern Methodist University
USNM: Smithsonian Institution National Museum of Natural History
ZPAL: Institute of Paleobiology (Zaklad Paleobiologii) of the Polish Academy of Sciences.

## Additional Information and Declarations

### Data Availability

The following information was supplied regarding data availability: No raw data was generated during this study.

### New Species Registration

The following information was supplied regarding the registration of a newly described species: Publication LSID: urn:lsid:zoobank.org:pub:6B5B8495-206C-4341-B728-6F77A0A1BA7C. Genus LSID: urn:lsid:zoobank.org:act:4B019789-0F7E-4C01-8C1D-7CAAA860CA26. Species LSID: urn:lsid:zoobank.org:act:2641A5F4-1EE4-4276-BB3A-247025557628.
