# Supplementary material for: Cranial osteology of the ankylosaurian dinosaur formerly known as Minmi sp. (Ornithischia: Thyreophora) from the Lower Cretaceous Allaru Mudstone of Richmond, Queensland, Australia
Source: PeerJ. 2015 Dec 8;3:e1475. doi: 10.7717/peerj.1475 (PMC4675105; doi:10.7717/peerj.1475)
Supplement: Appendix S1 [file peerj-03-1475-s001.pdf]

Appendix 1. Cranial Measurements

All measurements where taken using electronic callipers and are maximum dimensions (mm). Bones that are single in one aspect but are paired in a different aspect are marked with an asterisk (*), broken bones marked with hash (#), bones not visible or missing in an aspect marked with a dash (-).

Cranial bone (single)	Aspect	Rostrocaudal (mm)	Lateral (mm)	Dorsoventral (mm)
Cranium (whole)	dorsal	251.00	195.18	-
	ventral	243.23	239.82	-
Basioccipital	caudal	-	30.29	20.79
	ventral	45.30	23.76	-
Basisphenoid	ventral	55.12	62.46	-
Parasphenoid	ventral	37.62	13.73	-
Parietal*	dorsal	45.70	102.53	-
Supraoccipital	caudal	-	32.14	24.75

Cranial bone (paired)	Aspect	Rostrocaudal (mm)		Lateral (mm)		Dorsoventral (mm)	
		Left	Right	Left	Right	Left	Right
Exoccipital	caudal	-	-	71.54	68.95	52.29	54.29
Frontal	dorsal	67.85	65.87	49.99	48.39	-	-
Jugal	lateral	93.24	#	-	-	51.30	#
	ventral	94.21	#	24.31	#	-	-
Lacrimal	lateral	10.25	#	-	-	72.20	#
Maxilla	lateral	88.90	#	-	-	42.82	#

	ventral	169.11	124.35	42.78	57.01	-	-
Nasal	dorsal	120.25	113.27	43.36	43.41	-	-
	lateral	78.15	53.93	-	-	44.26	58.52
	rostral	-	-	43.36	43.41	49.85	60.22
Parietal*	caudal	-	-	47.87	45.00	30.44	30.25
Prefrontal	dorsal	74.67	76.55	41.52	42.42	-	-
Premaxilla	lateral	59.14	34.50	-	-	53.50	45.33
Postorbital	dorsal	59.39	56.51	63.85	65.11	-	-
	lateral	66.47	60.21	-	-	59.52	#
Pterygoid	ventral	46.89	50.63	46.75	35.12	-	-
Pterygoid (rostral process)	ventral	47.61	61.01	15.61	19.11	-	-
Quadrate	caudal	-	-	36.16	-	50.87	-
	ventral	11.48	90.00	40.52	26.46	-	-
Quadratojugal	ventral	49.34	-	11.07	-		
Squamosal	dorsal	13.79	15.91	40.19	43.48	-	-
	lateral	-	-	-	-	-	#
	caudal	-	-	35.37	35.75	26.41	28.30
Supraorbital	dorsal	57.91	51.42	33.65	34.05	-	-
	lateral	58.92	60.46	27.28	24.51	-	-

Appendix 2. Skull material examined firsthand

Ankylosaurus magniventris (AMNH 5214, 5895; TMP 66.2.02, cast; viewed at OU)

Edmontonia longiceps (AMNH 3076; CMN 8531 cast; viewed at NHMUK)

Edmontonia rugosidens (AMNH 5381; AMNH 5665)

Euoplocephalus tutus (AMNH 5223, 5337, 5403, 5404, 5405; NHMUK R4947)

Gastonia burgei (CEUM 1307 and BYU 14611)

Hungarosaurus tormai (MTM 2007.26.1–2007.26.34, 2007.89.1, 2007.89.2 (originally described as MTM Gyn 404), MTM 2007.25.1–2007.25.30; MTM V.2003.12; MTM 2007.26.4)

Pawpawsaurus campbelli (SMU 73203, cast; viewed at OU)

Pinacosaurus grangeri (AMNH 6523; ZPAL MgD-II/1; IGM 100/1014)

Pinacosaurus sp. (cast viewed at OU)

Sauropelta edwardsi (AMNH 3035)

Scelidosaurus harrisonii (NHMUK R1111)

Silvisaurus condrayi (KUVP 10296 cast; viewed at the NHMUK)

Tarchia gigantea (PIN 3142/250 cast)

Tarchia kielanae (INBR 21004, cast; viewed at OU) previously *Minotaurasaurus* (Arbour, Currie & Badamgarav et al. 2014).

Institutional abbreviations. AM, Australian Museum, Sydney, New South Wales, Australia; AMNH, American Museum of Natural History, New York City, New York, USA; BYU, Earth Science Museum, Brigham Young University, Provo, Utah, USA; CEUM, Prehistoric Museum, Utah State University, Price, Utah, USA (formerly the College of Eastern Utah Prehistoric Museum); CM, Carnegie Museum of Natural History, Pittsburgh, Pennsylvania, USA; CMN, Canadian Museum of Nature, Ottawa, Ontario, Canada (formerly the National Museum of Canada); IGM, Institute of Geology, Ulaan Baatar, Mongolia; IMM, Inner Mongolian Museum, Hohhot, China; INBR, Victor Valley Museum, Apple Valley, California, USA; IVPP, Institute

for Vertebrate Palaeontology and Paleoanthropology, Beijing, China; KUVP, Museum of Natural History, University of Kansas, USA; MTM, Magyar Természettudományi Múzeum (Hungarian Natural History Museum), Budapest, Hungary; NHMUK, Natural History Museum, London, England (formerly BMNH, British Museum of Natural History); OU, Ohio University, Athens, Ohio, USA; PIN, Paleontological Institute, Moscow, Russia; ROM, Royal Ontario Museum, Toronto, Canada; QM, Queensland Museum, Brisbane, Queensland, Australia (QM F, fossil; QM L, location); SMU, Shuler Museum of Paleontology, Southern Methodist University, Dallas, Texas, USA; TMP, Royal Tyrrell Museum of Palaeontology, Drumheller, Alberta, Canada; ZPAL, Institute of Paleobiology (Zaklad Paleobiologii) of the Polish Academy of Sciences, Warsaw, Poland.
