# Supplementary figures and images for: Cranial osteology of the ankylosaurian dinosaur formerly known as Minmi sp. (Ornithischia: Thyreophora) from the Lower Cretaceous Allaru Mudstone of Richmond, Queensland, Australia
Source: PeerJ. 2015 Dec 8;3:e1475. doi: 10.7717/peerj.1475 (PMC4675105; doi:10.7717/peerj.1475)

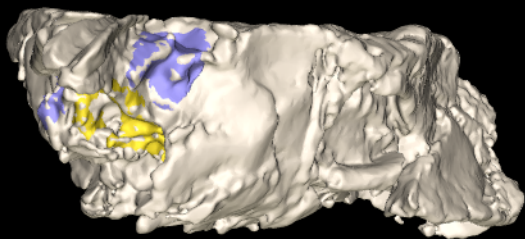

Supplement: Supplemental Information 1 [file peerj-03-1475-s002.pdf]
